# Modelled mortality benefits of multi-cancer early detection screening in England

**DOI:** 10.1038/s41416-023-02243-9

**Published:** 2023-04-25

**Authors:** Peter Sasieni, Rebecca Smittenaar, Earl Hubbell, John Broggio, Richard D. Neal, Charles Swanton

**Affiliations:** 1 Comprehensive Cancer Centre, King’s College London, Guy’s Campus, Great Maze Pond, London, SE1 1UL UK; 2 GRAIL Bio UK Ltd, a subsidiary of GRAIL, LLC, London, WC1V 7HP UK; 3 GRAIL, LLC, Menlo Park, CA 94025 USA; 4 NHS Digital, 7 and 8 Wellington Place, Leeds, West Yorkshire LS1 4AP UK; 5 Department of Health and Community Sciences, Faculty of Health and Life Sciences, University of Exeter, Exeter, UK; 6 Cancer Research UK Lung Cancer Centre of Excellence, University College London Cancer Institute, London, WC1E 6DD UK; 7 Cancer Evolution and Genome Instability Laboratory, Francis Crick Institute, London, NW1 1AT UK

**Keywords:** Cancer epidemiology, Cancer screening

## Abstract

**Background:**

Screening programmes utilising blood-based multi-cancer early detection (MCED) tests, which can detect a shared cancer signal from any site in the body with a single, low false-positive rate, could reduce cancer burden through early diagnosis.

**Methods:**

A natural history (‘interception’) model of cancer was previously used to characterise potential benefits of MCED screening (based on published performance of an MCED test). We built upon this using a two-population survival model to account for an increased risk of death from cfDNA-detectable cancers relative to cfDNA-non-detectable cancers. We developed another model allowing some cancers to metastasise directly from stage I, bypassing intermediate tumour stages. We used incidence and survival-by-stage data from the National Cancer Registration and Analysis Service in England to estimate longer-term benefits to a cohort screened between ages 50–79 years.

**Results:**

Estimated late-stage and mortality reductions were robust to a range of assumptions. With the least favourable dwell (sojourn) time and cfDNA status hazard ratio assumptions, we estimated, among 100,000 screened individuals, 74 (17%) fewer cancer deaths per year corresponding to 1787 fewer deaths in those screened between ages 50–79 years.

**Conclusion:**

Realising the potential benefits of MCED tests could substantially reduce late-stage cancer diagnoses and mortality.

## Introduction

Cancer caused 10 million deaths worldwide in 2020 [1]. It is the leading cause of death in most high income countries [2] including England, where it resulted in ~140,000 deaths in 2020 in a population of ~57 million [3, 4]. Early cancer detection is strongly associated with more treatment options for patients, leading to improved survival [5].

A key commitment of the National Health Service (NHS) in England’s Long Term Plan is to ensure that by 2028, 75% of cancers are diagnosed early, defined as at stage I or II [6]. Despite this, early cancer diagnosis has remained low, at ~54% [7]. England currently has nationally organised and quality-assured cancer screening programmes, all of which are free at the point of care, for bowel, breast, and cervical cancer, and is currently in the process of introducing a lung cancer screening programme. However, most cancers remain unscreened; over 80% of cancer deaths in the UK can be attributed to cancer types for which no population screening is currently available [8]. This gap suggests that innovations such as multi-cancer early detection (MCED) tests are needed to meaningfully improve cancer outcomes.

A screening programme using an MCED test has greater scope for benefits beyond single-cancer screening [9]. The low prevalence of individual cancer types makes it hard to develop single-cancer screening strategies in which the benefits outweigh the potential harms and costs. MCED tests are designed to test for multiple types of cancer with a fixed false-positive rate, in contrast to false-positive rate accumulation across multiple different single-cancer screening tests [10]. One blood-based MCED test (Galleri^®^) utilises circulating cell-free DNA (cfDNA) methylation patterns to detect a shared cancer signal from more than 50 cancer types and predict the cancer signal of origin (CSO) [11]. MCED screening has the potential to be a clinically- and cost-effective addition to the current screening programmes in the UK [12]. The randomised controlled NHS-Galleri trial (NCT05611632) aims to evaluate the clinical utility of this MCED test in an asymptomatic population [13].

Previous modelling demonstrated that a single screen with sensitivity as estimated in a case-control study [11] could result in 177–220 fewer late-stage diagnoses per 100,000 persons (there are currently 409 without MCED screening), corresponding to 74–91 fewer cancer deaths per 100,000 persons within five years (currently 393 without MCED screening), depending on modelling assumptions [14]. Recent research suggests that DNA shedding may be a marker of tumour aggressiveness [15]. Here, we built on this work to develop a model which (1) considers the differential survival of cfDNA-detectable (cfDNA^+^; i.e. shedding) cancers versus cfDNA-non-detectable (cfDNA^–^) cancers, and (2) is well-calibrated to current population cancer incidence and survival data in England, permitting an exploration of potential late-stage reduction and mortality benefits of MCED-based screening in England. We considered both the immediate impact of screening in a mixed-age cohort and the long-term impact in a cohort 50 years of age who were ‘aged’ through annual screening until they were 79 years old (national screening programme).

## Methods

### Data

This study used data provided by patients and collected by the NHS as part of their care and support. The data are collated, maintained and quality-assured by the National Cancer Registration and Analysis Service, which is part of NHS Digital [16, 17]. Crude incidence rates and net survival were calculated for 24 main cancer types and an additional ‘other’ category (see Table S1 for definitions) based on 50–79-year-olds diagnosed with cancer between 2013 and 2018 for each stage and by five-year age band. The age range is aligned with the 50–77-year age criterion for enrolment in the NHS-Galleri trial (NCT05611632) [13], allowing an additional two years of screening while enroled in the trial; this reflects the intended roll-out age group of an annual screening programme. For the purposes of the model, lymphoid leukaemia, myeloid neoplasm and plasma cell neoplasm were considered to be unstageable and there is no modelled mortality advantage from early detection. Incidence rates per 100,000 persons were provided for all cancers, apart from sex-specific cancers (ovary, cervix, uterus, prostate) for which person rates were created by multiplying the rate by the proportion of the relevant sex in the five-year age band. Age-specific net survival was estimated using a period approach with the Pohar Perme estimator [18], capped at 99.9%. The analysis was censored on 5 January 2020, providing a minimum of one year of follow-up for all patients. Aggregate incidence and survival data used in the analysis are provided in Tables S2, S3.

### Interception model

**Fig. 1:**
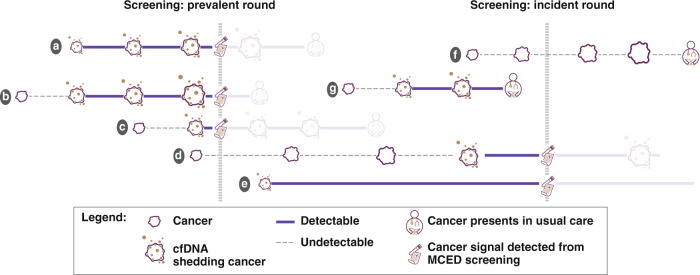
Interception model schematic. Before a single prevalent round of screening, cancer **a** was shedding cell-free DNA (cfDNA) at stage I and progressed to stage III, when it was intercepted; **b** became cfDNA shedding at stage II and progressed to stage IV, when it was intercepted; **c** became cfDNA shedding at stage II, when it was intercepted; **d** was not shedding cfDNA at stage I when it was screened in a prevalent screening round, and progressed to stage III before it was intercepted in the incident screening round; **e** became cfDNA shedding at stage I and did not progress before being intercepted at the incident screening round; **f** never became cfDNA-detectable, was not intercepted by MCED screening, and presented in usual care at stage IV; and **g** became cfDNA-detectable at stage II and progressed to stage III between screening rounds, when it was detected via usual care.

Hubbell and colleagues [14] first presented the interception model (Fig. 1). We extended this model to (1) accommodate emerging evidence regarding the prognostic significance of cfDNA^+^ versus cfDNA^–^ cancers [19] and (2) estimate the impact of an annual national screening programme (30 years of screening in a cohort aged 50 years until 79 years of age) by incorporating attrition from the programme due to mortality and cancer diagnosis. Additionally, we completed a sensitivity analysis to model the impact of non-sequential stage progression (varying proportions of tumours metastasising early, with direct progression from stage I to IV, for a subset of aggressive cancers) by reducing stage II and III dwell times to zero (Analysis S4).

The interception model starts by considering a population of incident cancers under usual care. We calculated the number of these cancers present (but undetected) at earlier stages in prior years using estimates of dwell (sojourn) times with exponential distributions. Dwell time corresponds to the amount of time a tumour spends in each stage before progressing to the next stage. Mean dwell times (for each stage in each cancer type) were estimated based on expert elicitation for the interception model published by Hubbell and colleagues [14]. In typical situations, cancers are treated shortly after diagnosis, therefore there is no reliable data on the time it takes for cancer to progress between stages. Dwell times cannot be inferred from cancer patients who are not treated, as these patients cannot be considered representative; other comorbid conditions and frailty complicate the clinical picture. Mean dwell times are therefore intrinsically uncertain, so we have chosen to model a range of scenarios. Lastly, we calculated the number of cancers intercepted at each stage, based on cancer type- and stage-specific sensitivity of an MCED test (Galleri^®^) which were estimated in a case-control study [11] and adjusted using isotonic regression so that test sensitivity did not decrease by stage (Table S5). Throughout the manuscript, we use the generic term MCED screening to reflect that this model could be used with different cancer type- and stage-specific sensitivity estimates, though the results estimated in this paper are based on Galleri^®^ test sensitivity estimates [11]. We used dwell times specific to each cancer type and stage, as previously described by Hubbell and colleagues [14], and the individual variation in cancers of the same type was modelled using an exponential distribution (Table S6).

Interception occurs if an individual is screened when a cancer is present and detectable, which in turn depends on the sensitivity of the test (Method S7). The detection of detectable cancers depends on the time interval between screening rounds relative to stage dwell times. The proportion of cancers that are intercepted increases as the interval between screening rounds is shortened, with a maximum proportion equal to stage-specific test sensitivity. Additionally, in modelled scenarios with longer (slower) dwell times, cancers are less likely to progress through stages in the window of time between screening rounds, and more are intercepted at earlier stages. For cancers that are unstageable, a single sensitivity value is assumed, from which the proportion of detected cancers is calculated. Therefore, these cancers do not contribute to the estimation of late-stage cancer reduction or consequent mortality, but only to the proportion of cancers detected via the MCED screening route. We imputed cancers with missing stage information by proportionately increasing the incidence in each stage. Given staging procedures are often not undertaken as it is anticipated the patient will not benefit from them, it is anticipated many missing stage cancers would be late-stage cancers. As such, this imputation is conservative as it reduces the number of late-stage cancers available to be intercepted. Results presented here are for a cohort of individuals who participate in screening. Diagnostic resolution was assumed to follow shortly after screen detection, and we did not model the impact of returning ‘cancer signal origin’ results to the diagnosing physician.

### Mortality modelling

After computing the stage shift based on the interception model, we estimated mortality benefits by comparing the difference in stage-specific survival between stage distributions with and without MCED screening in addition to usual care. Specifically, for instance, a breast cancer that would have been clinically diagnosed at stage IV, following interception at stage II, would be assigned the survival of a stage II breast cancer. This within-cancer-type modelling approach allows for prognostic heterogeneity between cancer types for different stages. We used a two-population model for survival, stratified by stage at diagnosis, to model differential survival depending on cfDNA detectability status (cfDNA^+^ or cfDNA^–^). Based on previous findings regarding the increased risk of death from cfDNA^+^ cancers [19], we modelled cfDNA^+^ cancers as having double (hazard ratio [HR] = 2) or triple (HR = 3) the stage-specific risk of cancer death compared with cfDNA^–^ cancers of the same cancer type. Net survival was adjusted for both cfDNA^+^ and cfDNA^–^ status to ensure that, given the population fraction of each cancer type at each stage, average survival would match that observed in the population, resulting in reduced mortality for cfDNA^–^ cases compared with population net survival-by-stage estimates. This adjustment was applied to all cfDNA^+^ cancers, intercepted or not; thus, in the resultant survival data for both cfDNA^+^ and cfDNA^–^, the total mortality in the overall population was preserved. For comparison, we also modelled a scenario similar to Hubbell and colleagues [14] in which survival for cfDNA^+^ and cfDNA^–^ cancers was identical (HR = 1).

For both survival populations, we modelled lead-time until date of diagnosis via usual care, during which individuals cannot die from their cancer (to avoid a situation in which early detection causes cancer death before a symptomatic tumour) [20]. We then applied the HR to net survival-by-stage to estimate cancer-specific mortality (so that expected mortality was calculated for years following diagnosis under usual care, regardless of interception occurrence). Expected cancer mortality within five years of the date of diagnosis under usual care was calculated per 100,000 persons screened. The difference in cancer mortality with MCED screening compared with usual care is the estimated mortality benefit with MCED screening.

### Modelled scenarios

#### Open cohort

This modelled scenario is comparable to the NHS-Galleri trial, run in a population attending their first (prevalent) screen at ages 50–79 years, followed by subsequent (incident) annual screening tests. This model assesses how the removal of previously intercepted cancers from the screened population impacts late-stage cancer incidence and cancer mortality rates.

#### National screening programme cohort

This modelled scenario enabled comparisons between the presence and absence of an MCED-based screening programme. For both incidence and survival data, participants entered the cohort at 50 years of age, and were ‘aged’ through the programme until they turned 80 years old, died, or emigrated, with different age-specific incidence rates and net survival probabilities applied in the interception model at each of the 30 annual screening rounds. Figure S8 shows the cohort weights applied at each age, corresponding to movement out of the cohort.

The cumulative benefits of this ‘national screening programme’ scenario were calculated using a life table to adjust for attrition from all-cause mortality [21] and a competing cancer diagnosis. The weighted average of male and female mortality was used to generate a mortality rate for all persons. Participants did not re-enter the screening cohort after a cancer diagnosis, even though many will survive their cancer diagnosis. This is a simplifying assumption; for some cancer types, cancer survivors are at greater risk for the development of subsequent cancers [22]. However, survival for some early-stage cancers (e.g. breast and prostate cancer) is very high, and people who survive these cancers may go on to develop a fatal cancer that is not captured in the model, because they have been censored out. This assumption equates the risk of developing a cancer in the population without a competing cancer diagnosis to that observed in the whole England population. It is beyond the scope of the current paper to consider these complexities of screening cancer survivors.

## Results

NCRAS data for England demonstrated an average annual cancer incidence of 1213 per 100,000 persons in 50–79 year olds in the period 2013–2018. Of these cancers, 1164 per 100,000 were stageable and 973 per 100,000 were staged, with 450 per 100,000 diagnosed at a late stage (III or IV).

### Open cohort model: overall late-stage incidence and mortality reductions

Table 1 shows the number of cancers (per 100,000 persons screened) found via usual care alone and when MCED screening was added to usual care, and the subsequent reduction in late-stage cancer incidence with the addition of MCED screening.

**Table 1:** Late-stage incidence reduction in the open cohort scenario.

		Dwell Time Scenario		
	No Screening	Slow	Medium	Fast
**Prevalent Screening Round**				
**Basic Performance** (Including Future Year Cancers Found Now)				
**Found Via Usual Care and MCED (%)**	NA	3768 (100)	2847 (100)	2150 (100)
**Found Via Usual Care (%)**	NA	1003 (27)	1056 (37)	1151 (54)
**MCED Detected (%)**	NA	2765 (73)	1791 (63)	999 (46)
**Late-Stage Reduction** (Including Future Year Cancers Found Now)				
**Late-Stage Diagnosis With Usual Care (%)**	NA	2401 (64)	1688 (59)	1128 (52)
**Late-Stage Diagnosis With MCED (%)**	NA	799 (21)	690 (24)	573 (27)
**Reduction in Late-Stage Diagnosis With MCED (%)**	NA	1602 (67)	999 (59)	555 (49)
**Incident Screening Round**				
**Basic Performance** (Steady State)				
**Found Via Usual Care and MCED (%)**	1192 (100)	1192 (100)	1192 (100)	1192 (100)
**Found Via Usual Care (%)**	1192 (100)	727 (61)	764 (64)	833 (70)
**MCED Detected (%)**	NA	465 (39)	428 (36)	359 (30)
**Late-Stage Incidence Reduction** (Steady State)				
**Late-Stage Diagnosis With Usual Care (%)**	506 (42)	506 (42)	506 (42)	506 (42)
**Late-Stage Diagnosis With MCED (%)**	NA	257 (22)	276 (23)	305 (26)
**Reduction in Late-Stage Diagnosis With MCED (%)**	NA	248 (49)	230 (45)	201 (40)

For the ‘open cohort’ modelled scenario, the impact of multi-cancer early detection (MCED) screening on the incidence rate of cancers diagnosed at a late stage (III and IV) is shown for each dwell time scenario and for prevalent and incident screening rounds. These results are directly comparable to estimates produced for introducing a screening programme based on an MCED test, with sensitivity as estimated in a case–control study (11), to the US population (14). All results are incidence rates per 100,000 persons screened. Prevalent rounds of screening were most susceptible to dwell time assumptions. This is reflected in the mortality rates without MCED screening because there are different numbers of cancers available to be diagnosed through usual care, depending on assumptions regarding the speed of dwell times. This is in contrast with the incident rounds of screening, in which the previous prevalent rounds ‘level-set’ the numbers of cancers available for diagnosis. As the model is focused on screening referral performance, we did not explicitly model the diagnostic resolution process, which may be extended due to work-up at multiple potential cancer signal origins, or terminated after a single diagnostic work-up, with the risk of missing a cancer. Instead, we made the simplifying assumption that diagnostic resolution occurs shortly after detection. Another simplifying assumption was that ‘usual care’ refers to the standard practice in terms of screening, primary care referral, diagnostic work-up and treatment resulting in the incidence-by-stage and survival-by-stage data observed in the period 2013–2018 in England. This assumption was to incorporate non-adherence to screening, diagnostic and treatment guidance, and limited access to healthcare. The denominator for the percentage calculations corresponds to all staged and unstageable cancers. Stageable cancers with missing stage information were excluded from the analysis. Data are to the nearest whole number; therefore, breakdowns of total numbers may not always sum perfectly.

When MCED screening was first added to usual care (prevalent round), cancers which would otherwise have been diagnosed in future years are intercepted, increasing incidence for that year. Per 100,000 individuals screened, 555 of the 1128 originally late-stage (III + IV) cancers (fast), and 1602 of the 2401 originally late-stage cancers (slow) were found instead at an early stage (Table 1). This indicates a strong influence of dwell times on the impact of screening in the prevalent round.

Incident screening rounds were less susceptible to changes in dwell times, and correspondingly we observed a narrower range in the number of cancers found via usual care with and without MCED screening (Table 1). The annual reductions in late-stage incidence with MCED screening added to usual care were predicted to be between 201 and 248 per 100,000 persons screened. These rates were considerably lower than in the prevalent screen, reflecting the fact that cancers diagnosed via screening are removed from the available population each year.

In Table 2 we present five-year mortality rates per 100,000 persons screened with and without MCED screening added to usual care, and the resulting mortality rate differences with MCED screening. The effect of different dwell times on mortality reduction in the prevalent screening round was estimated to be greater than the effect of different HRs for cfDNA^+^ versus cfDNA^–^ cancers. The estimated mortality rate reduction when MCED screening was added to usual care was between 211 (fast dwell time, HR = 3) and 686 (slow dwell time, HR = 1) deaths per 100,000 persons screened in the prevalent screening round (Table 2), and between 74 (fast dwell time, HR = 3) and 107 (slow dwell time, HR = 1) deaths per 100,000 persons in the incident screening round (Table 2).

**Table 2:** Five-year mortality rate reduction in the open cohort scenario.

	No Screening	Hazard Ratio = 1			Hazard Ratio = 2			Hazard Ratio = 3		
		Slow	Medium	Fast	Slow	Medium	Fast	Slow	Medium	Fast
**Prevalent Screening Round (Including Future Year Cancers Found Now)**										
**Cancer Mortality Rate With Usual Care**	NA	1954	1375	964	2007	1405	975	2036	1420	981
**Cancer Mortality Rate With MCED**	NA	1268	941	714	1386	1010	750	1451	1048	770
**Reduction in Cancer Mortality Rate With MCED, (%)**	NA	686 (35)	434 (32)	250 (26)	621 (31)	395 (28)	225 (23)	585 (29)	372 (26)	211 (22)
**Incident Screening Round (Steady State)**										
**Cancer Mortality Rate With Usual Care**	442	442	442	442	442	442	442	442	442	442
**Cancer Mortality Rate With MCED**	NA	335	343	355	346	352	363	352	358	368
**Reduction in Cancer Mortality Rate With MCED (%)**	NA	107 (24)	99 (22)	87 (20)	96 (22)	90 (20)	79 (18)	90 (20)	84 (19)	74 (17)

For the ‘open cohort’ modelled scenario, the impact of screening with a multi-cancer early detection (MCED) test with sensitivity as estimated in a case–control study (11) on the five-year cancer mortality rate is shown per 100,000 persons screened. Results are displayed for each dwell time scenario and for differential survival hazard ratios (HRs) for cell-free DNA^+^ (cfDNA^+^) cancers versus cfDNA^–^ cancers. HR = 1 corresponds to no increased risk of mortality with a cfDNA^+^ cancer. Prevalent rounds of screening are most susceptible to dwell time assumptions, as reflected in the late-stage incidence reduction results in Table 1. There is additional complexity in that different HRs contribute to different mortality rates between scenarios, even without MCED screening, because cfDNA^+^ cancers are present irrespective of whether or not they are intercepted. The data should be interpreted as follows: for the fast dwell time scenario, there were 1717 cancers diagnosed in the prevalent round of screening (Table 1), and assuming HR = 1, there were 770 deaths from cancers diagnosed via usual care, and 570 deaths from cancers diagnosed when MCED screening was added to usual care. This corresponds to a difference in the five-year cancer mortality rate of 200 per 100,000 persons screened (26%) due to the addition of MCED screening to usual care. Data are to the nearest whole number; therefore, breakdowns of total numbers may not always sum perfectly.

### Open cohort model: late-stage incidence and mortality rate reductions by cancer type

The potential late-stage incidence reduction benefit varied by cancer type (Table 3). For colon/rectum, head and neck, liver/bile duct, lung, lymphoma, ovary and pancreas cancers (Table 3), the reduction in late-stage incidence with MCED screening was estimated to be substantial (>10 per 100,000 persons screened), reflecting high test sensitivity and current adverse stage profiles for these cancers. Although breast cancer is predominantly diagnosed at an early stage, the reduction of 9 per 100,000 in late-stage incidence reflects the potential to diagnose this high-incidence cancer at stage I or II, despite the relatively poor sensitivity at earlier stages.

**Table 3:** Late-stage incidence and five-year mortality rate reductions by cancer type.

	Current Incidence Rate	Late-Stage Diagnosis With Usual Care	Late-Stage Diagnosis With MCED	Reduction in Late-Stage Diagnosis With MCED (%)	Cancer Mortality Rate With Usual Care	Cancer Mortality Rate With MCED	Reduction in Mortality With MCED (%)
**Colon/Rectum**	129	71	24	47 (66)	45	21	25 (56)
**Lung**	155	113	49	64 (57)	126	108	18 (14)
**Ovary**	26	17	5	12 (71)	14	6	7 (50)
**Head and Neck**	42	28	7	22 (79)	16	8	8 (50)
**Breast**	176	23	14	9 (39)	14	10	4 (29)
**Liver/Bile Duct**	22	15	4	11 (73)	18	14	4 (22)
**Pancreas**	32	25	11	14 (56)	30	27	3 (10)
**Oesophagus**	31	23	12	10 (43)	24	22	2 (8)
**Lymphoma**	50	34	17	17 (50)	14	11	3 (21)
**Prostate**	195	81	77	3 (4)	16	14	2 (12)
**Bladder**	29	8	6	1 (12)	12	10	2 (17)
**Cervix**	5	2	0	1 (50)	2	1	1 (50)
**Kidney**	37	16	14	2 (12)	11	10	1 (9)
**Sarcoma**	9	4	2	2 (50)	4	3	1 (25)
**Stomach**	18	13	8	5 (38)	14	13	1 (7)
**Uterus**	34	6	4	2 (33)	6	5	1 (17)
**Anus**	5	3	1	2 (67)	1	1	0 (0)
**Gallbladder**	7	4	4	1 (25)	5	5	0 (0)
**Melanoma**	51	5	5	0 (0)	4	4	0 (0)
**Thyroid**	8	4	4	0 (0)	1	1	0 (0)
**Urothelial Tract**	6	4	4	0 (0)	3	3	0 (0)

For adults aged 50–79 years, for each cancer type, the table shows: crude incidence rates (per 100,000 persons); incidence rates for late-stage (III & IV) cancer diagnoses with usual care, and with multi-cancer early detection (MCED) screening (with sensitivity as estimated in a case–control study [11]) added to usual care; incidence rates of cancers shifted early by MCED screening; five-year mortality rate reductions (per 100,000 persons screened) for usual care, and with MCED screening added to usual care. For mortality rates, we used a survival hazard ratio (HR) of three for cell-free DNA^+^ cancers versus cfDNA^–^ cancers, to provide the most conservative estimate of benefit from MCED screening. Data are to the nearest whole number; therefore, breakdowns of total numbers may not always sum perfectly. The table has been sorted by absolute reduction in mortality with MCED screening.

In contrast, MCED screening did not appear to contribute to earlier diagnosis for melanoma, thyroid and urothelial tract cancers, and for prostate cancers, the proportion of cancers shifted from late to early stage was small. This reflects both lower MCED test sensitivity at early stages for these cancers and that the majority of them are already diagnosed at an early stage.

For breast, colon/rectum, head and neck, liver/bile duct, lung, lymphoma, ovary and prostate, sarcoma and uterus cancers, the reduction in late-stage incidence translates into >10% reductions in the five-year cancer mortality rate (deaths from cancer within five years of diagnosis, based on when the diagnosis would have happened in the absence of MCED screening), which reflects that early-stage survival is considerably better than late-stage survival for these cancer types. The substantial modelled mortality reduction for prostate cancer in the context of minimal late-stage incidence reduction highlights that the majority of prostate cancer mortality is observed among those diagnosed at stage IV, and therefore the impact of intercepting these cancers prior to progression to stage IV is proportionally large. For several relatively low-incidence cancer types including ovary, liver/bile duct, pancreas and oesophagus, the absolute number of deaths avoided with MCED is small; however, the proportional mortality benefits for these cancer types are substantial, as is the aggregate total mortality rate reduction of these low-incidence cancer types combined.

**Fig. 2:**
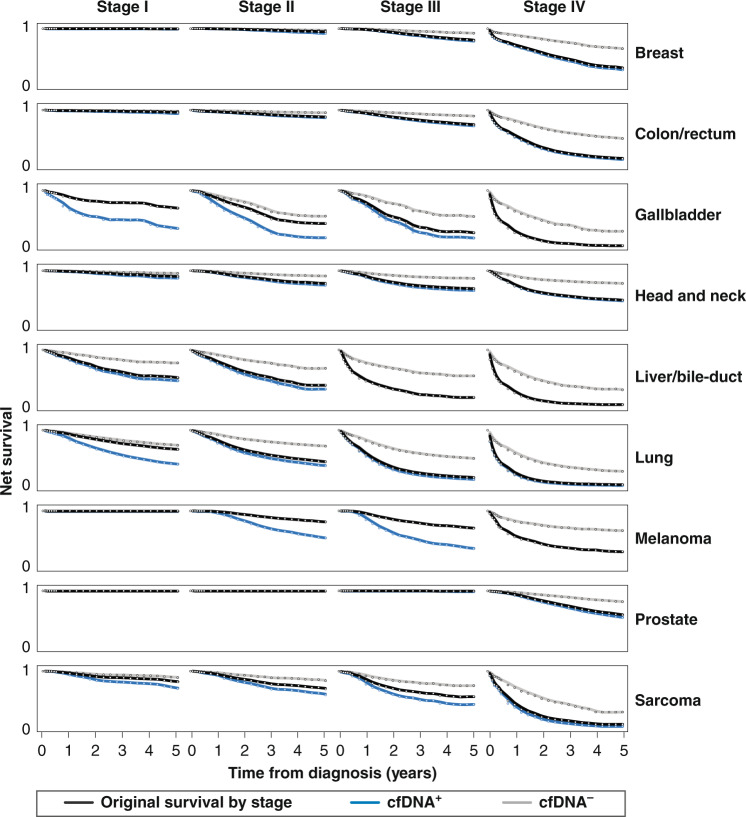
Survival by cfDNA status and stage for selected cancer types. This figure shows observed five-year survival data for cancers diagnosed between 2013 and 2018, as registered by the National Cancer Registration and Analysis Service (NCRAS), and modelled five-year survival data based on cfDNA detectability status (hazard ratio [HR] = 3). For visualisation purposes, we plotted net survival for a 65-year-old in the middle of the screening programme, and data has been smoothed. Where the lines representing cfDNA^+/−^ survival are not visible, they are (almost) identical to that of original survival. For a small number of cancers at specific stages, adjustment of survival data for both cfDNA-detectable and cfDNA-non-detectable cases to reflect the observed survival in the population created implausible results, in which later-stage survival for cfDNA-detectable cancers was favourable compared with that of earlier-stage cancers. This occurred when there was a large difference in test sensitivity between one stage and the next, such that applying the HR to the different cfDNA populations moved the survival curves further out from the observed mean. We did not correct for this anomaly, as it was only ever a difference of a few percentage points, and it would have decreased the average observed survival-by-stage.

For a subset of cancers stratified by stage, Fig. 2 shows modelled five-year survival for each cfDNA detectability status when HR = 3, along with observed survival based on NCRAS data. By cancer type and stage, when test sensitivity is either very low or high, the impact of having cfDNA^+^ status on survival is minimal. This is because the majority of cancers are cfDNA^+^ or cfDNA^–^, such that the modelled survival curves do not separate markedly from observed survival. The impact of low test sensitivity is demonstrated in early-stage breast and prostate cancers, for which all three survival curves closely overlap. For cancer types in which the majority of cancers are cfDNA^+^ even at early stages, such as liver/bile duct cancers and lung cancer from stage II onwards, the impact of cfDNA^+^ status is also minimal, as the modelled survival for these cancers closely follows observed survival. Conversely, the modelled survival for cfDNA^–^ cancers at these sites is considerably better, as almost all of the deaths over the period are among those with cfDNA^+^ cancers. The impact of cfDNA detectability status is more critical when the MCED test has a less extreme sensitivity, therefore it adds more prognostic information at a population level. This pattern is observed in stage II and III gallbladder cancer and sarcoma. Figure S9 shows the impact of cfDNA status by cancer type for all cancers included in this study.

### National screening programme: late-stage incidence and mortality rate reductions

In the national screening programme modelled scenario (Table 4), the incidence of cancer diagnosed through MCED screening was estimated to be between 8706 and 11,614 per 100,000 persons (for fast and slow dwell time assumptions, respectively), corresponding to a proportion of between 30 and 40% of cancers detected through MCED screening. Thus, the estimated reduction in the incidence rate of late-stage cancer was between 4865 and 6217 per 100,000 persons in the screening programme (40–50%). The reduction in five-year cancer mortality was between 1787 and 2257 per 100,000 persons in the screening programme (between 17 and 21%) in the fast dwell time scenario with HR = 3 and slow dwell time scenario with HR = 1, respectively. Figure S10 demonstrates how the incidence rate of MCED screen-detected cancers changes with age for each of the dwell time scenarios.

**Table 4:** Late-stage incidence and five-year mortality rate reductions in a national screening programme scenario.

Late-Stage Incidence and Five-Year Mortality Rate Reductions, National Screening Programme (Per 100,000 Persons Entering Screening Programme)			
Dwell Time Scenario	Slow	Medium	Fast
**Basic Performance**			
**Found Via Usual Care (%)**	17,412 (60)	18,313 (64)	19,947 (70)
**Found Via MCED (%)**	11,614 (40)	10,515 (36)	8706 (30)
**Late-Stage Incidence Reduction**			
**Late-Stage Diagnosis With MCED (%)**	6249 (24)	6666 (25)	7327 (28)
**Reduction in Late-Stage Diagnosis with MCED (%)**	6217 (50)	5658 (46)	4865 (40)
**Five-Year Cancer Mortality Rate**			
**HR cfDNA = 1**			
**Cancer Mortality Rate With Usual Care (%)**	8123 (28)	8265 (29)	8517 (30)
**Reduction in Cancer Mortality Rate With MCED (%)**	2664 (25)	2437 (23)	2115 (20)
**HR cfDNA = 2**			
**Cancer Mortality Rate With Usual Care (%)**	8393 (29)	8508 (30)	8729 (30)
**Reduction in Cancer Mortality Rate with MCED (%)**	2404 (22)	2199 (21)	1905 (18)
**HR cfDNA = 3**			
**Cancer Mortality With Rate Usual Care (%)**	8544 (29)	8645 (30)	8848 (31)
**Reduction in Cancer Mortality Rate With MCED (%)**	2257 (21)	2064 (19)	1787 (17)

This table shows cumulative benefits of a national screening programme based on a multi-cancer early detection (MCED) test, with sensitivity as estimated in a case–control study (11), starting from 50 years of age with annual screening until 79 years of age for three dwell time scenarios; mortality rate reduction outcomes are presented for three cfDNA hazard ratio (HR) scenarios. Data are to the nearest whole number; therefore, breakdowns of total numbers may not always sum perfectly.

Results of the non-sequential stage progression model (Supplementary Analysis S6) demonstrate that the benefits of MCED screening may be reduced in scenarios where greater proportions of cancers show non-sequential progression and have a shorter dwell time in stage I.

## Discussion

In this study, we extended the interception model first described by Hubbell et al. [14], to explore the benefits of MCED screening, with sensitivity as estimated in a case-control study [11], under a range of model assumptions. Overall, the reduction in late-stage cancer incidence and mortality rates were substantial under a range of assumptions regarding dwell times and differential survival between cfDNA-detectable and non-detectable cancer. Even with the fastest dwell times, the proportion of cancers screen-detected with MCED was estimated to be 31%. Given that in England, only ~6% of cancers are currently diagnosed via screening [23], widespread adoption of MCED screening among 50–79 year olds would improve the cancer diagnostic landscape. This has the potential to contribute to the NHS Long Term Plan goal of achieving 75% of cancers diagnosed at an early stage [6]; our model estimates that 49–56% of cancers are diagnosed at an early stage via usual care, which could be increased to 73–79% (74–78% in incident rounds of screening) among those who are screened. MCED screening (based on parameters of one commercially available test) has the greatest potential to reduce late-stage diagnoses and consequent mortality among cancers currently diagnosed predominately at late stages, and for which the MCED test has high sensitivity. Thus, MCED screening may contribute to the early detection, and, potentially, mortality reduction of cancers for which there is substantial unmet need. Even achieving the lower bound of mortality reduction via MCED screening would mean that cancer was no longer the leading cause of mortality among screened individuals in England [24].

The areas of uncertainty in the modelling will be resolved, in part, by the results of the NHS-Galleri trial, which will directly estimate the reduction in the incidence of late-stage cancers resulting from annual MCED screening in 50–79 year olds across three annual screening rounds [13]. Beyond late-stage incidence reduction, the trial results will help to inform model structure and parameter values in future iterations of the model presented here, which could in turn be used to extrapolate from NHS-Galleri trial results. Should the interception model be well-calibrated to the trial outcomes, it could be used to examine scenarios not directly tested in the trial, including different screening intervals and age cut-offs for the screening programme.

Results from the NHS-Galleri trial, as well as other emerging evidence, may also challenge structural aspects of the model. For example, non-sequential stage progression may be more common than previously thought [25]. If this is the case, then other credible alternative model structures, such as the non-sequential stage progression model detailed in the Supplementary Information (Analysis S10), may provide a better fit to the trial data. This model demonstrates that greater proportions of cancers showing non-sequential progression and a short (6 month) dwell time in stage I have the potential to reduce the benefits of MCED screening.

Chabon et al. [26] found that in lung cancer, cfDNA^+^ cancers are enriched for subclinical micrometastases that are invisible to current scanning techniques, suggesting that cfDNA^+^ cancers may be more lethal than cfDNA^–^ cancers, irrespective of their apparent stage at diagnosis. Here we estimated that MCED screening is beneficial even when cfDNA^+^ cancers had a three-fold greater relative mortality risk. However, longer-term follow-up is required to fully characterise the consequences of cfDNA detectability, both in case-control and screening settings in which these cancers are shifted to an earlier stage. There are no current data to suggest that cfDNA^+^ cancers are incurable, and there is some evidence to suggest that ~40% of circulating tumour DNA^+^ (ctDNA^+^) early-stage (I–III) non-small-cell lung cancers diagnosed through usual care remain free from recurrence up to five years after the end of treatment [27]. For breast, colorectal and lung cancer, cfDNA concentration has been linked to both tumour volume and surrogate biomarkers of clinically significant prognosis, including depth of invasion (colorectal), mitotic (breast), and metabolic activity [15], and adjuvant chemotherapy resistance (lung) [28]. This suggests inter-cancer heterogeneity in the mechanisms by which cfDNA enters the tumour microenvironment and subsequently the bloodstream (for a review of the issue, see ref. [29]). A greater understanding of these mechanisms is required to understand the extent to which cfDNA^+^ cancers are likely to be treatment-resistant, and how this corresponds to outcome heterogeneity. Further clinical trials may also be required to examine the efficacy of alternative or escalated systemic anti-cancer therapy regimens for such cancers, and/or de-escalated treatment regimens for cfDNA^–^ cancers. In this case, the MCED test may confer additional benefits as a risk stratification tool. Preliminary evidence also supports the use of ctDNA as a marker for molecular residual disease, to help identify when further treatment may help avoid recurrence [25, 27].

The performance of the MCED test was previously estimated in case-control settings to have a test sensitivity of 51.5% [11]. In a population screening trial such as the NHS-Galleri trial, in which participants are invited from the community, the performance of the test may differ. If cancers tend to shed cfDNA only shortly before becoming symptomatic, or if cfDNA-shedding cancers progress very rapidly, then patients would need to be tested more frequently to diagnose cancers earlier, limiting the test’s utility as a population-level screening tool. However, there is currently no evidence directly linking cfDNA shedding to disease progression or symptom development. Indeed, for lung cancer, detectable levels of cfDNA may be reached in some patients prior to tumour sizes that would likely result in symptomatic presentation [30].

Unlike conventional screening techniques, especially those based on imaging or direct visualisation, the MCED screening signal is based on cancer biology; thus, overdiagnosis (of indolent lesions) is less of a concern, particularly compared with multiple separate conventional screening techniques for individual cancers, in which the risks associated with each screening mode accumulate [31]. Poor sensitivity to cancers with known overdiagnosis issues, including early-stage breast, prostate, and thyroid cancers [32], provides reassurance that MCED screening will not contribute to overdiagnosis. As such, overdiagnosis was not incorporated in the current model. However, this is the first time that a candidate screening technology has existed for most cancer types, and the understanding of cfDNA is nascent. The possibility of overdiagnosis will be investigated in the NHS-Galleri trial [13], which may inform the evidence base on the natural history of cancer. Future iterations of the model will incorporate these considerations.

We used five-year net survival from the date of usual-care diagnosis in this study [33], but some cancer patients are still at increased risk of death ten years after their initial diagnosis [34]. While a longer follow-up period may allow estimation of survival over ten or more years, survival with more historical cancer cases would not capture current best treatment practices, and the availability of historic staging data is limited.

Additionally, we only considered an idealised 100% screening attendance scenario with rapid subsequent diagnostic resolution. The harms associated with screening such as psychological distress, diagnostic work-up of false-positive results, and healthcare resource utilisation should be considered in future studies on real-world attendance, and the feasibility and implications of national MCED screening.

Some other potential benefits of MCED screening are not captured in the study. MCED screening may lead to within-stage changes in cancer detection that are prognostically relevant and confer mortality benefits, but do not lead to stage shift. There are treatments for patients with oligometastatic lesions with the potential for long-term survival that are not an option for patients with widely disseminated metastases [35–40]. Within-stage reduction in breast tumour size due to screening has been shown to be associated with clinically relevant reductions in mortality [41]. Diagnosis via a managed (rather than emergency) pathway is also associated with improved survival [42]. A higher level of granularity in the anticipated prognosis could be gained by including additional tumour characteristics or substages in the model. We accounted for this to some extent by allowing for differential stage-specific hazards in cfDNA^+^ and cfDNA^–^ cancers, but the model could be modified with further empirical data. Lastly, potential morbidity benefits to patients were not considered in this study. Cancers curable at a late stage may be shifted to earlier stages with MCED screening, and patients with these cancers are likely to experience quality of life benefits from less toxic treatments, even if no mortality benefit is observed [43].

In summary, we built upon the interception model to incorporate recent evidence regarding the prognostic significance of cfDNA detectability status and estimate the longer-term benefits of a national screening programme in England, from where the NHS-Galleri trial has enroled participants. Despite uncertainty regarding this intervention, we demonstrated that the potential benefits are robust to a range of model parameters, and that MCED screening may contribute to reduction in the incidence of late-stage cancers and consequent mortality, particularly for cancers with a current adverse staging profile. The magnitude of the benefits estimated here supports the launch of the NHS-Galleri trial.

## Supplementary information

Supplementary Information

## Data Availability

These data can be made publicly available on request.

## Code availability

The code used to generate the results in this study are available on request from the corresponding author.

## References

[1] Sung H, Ferlay J, Siegel RL, Laversanne M, Soerjomataram I, Jemal A, et al. Global cancer statistics 2020: GLOBOCAN estimates of incidence and mortality worldwide for 36 cancers in 185 countries. CA Cancer J Clin. 2021;71:209–49. 33538338 10.3322/caac.21660

[2] Bray F, Ferlay J, Soerjomataram I, Siegel RL, Torre LA, Jemal A. Global cancer statistics 2018: GLOBOCAN estimates of incidence and mortality worldwide for 36 cancers in 185 countries. CA Cancer J Clin. 2018;68:394–424. 30207593 10.3322/caac.21492

[3] Office for National Statistics. Population estimates. https://www.ons.gov.uk/peoplepopulationandcommunity/populationandmigration/populationestimates. Accessed 22 November 2022.

[4] Office for National Statistics. Total cancer deaths in the UK in 2019 and 2020. https://www.ons.gov.uk/aboutus/transparencyandgovernance/freedomofinformationfoi/totalcancerdeathsintheukin2019and2020. Accessed 23 November 2022.

[5] NHS Digital. Cancer survival in England, cancers diagnosed 2015 to 2019, followed up to 2020. https://digital.nhs.uk/data-and-information/publications/statistical/cancer-survival-in-england/cancers-diagnosed-2015-to-2019-followed-up-to-2020. Accessed 22 November 2022.

[6] National Health Service. The NHS long term plan. https://www.longtermplan.nhs.uk/wp-content/uploads/2019/08/nhs-long-term-plan-version-1.2.pdf. Accessed 22 November 2022.

[7] National Cancer Registration and Analysis Service. Cancer data: Percentage of stageable cancers diagnosed at stage 1 and 2 in England. https://www.cancerdata.nhs.uk/stage_at_diagnosis. Accessed 22 November 2022.

[8] Cancer Research UK. Cancer mortality statistics. https://www.cancerresearchuk.org/health-professional/cancer-statistics/mortality/common-cancers-compared. Accessed 22 November 2022.

[9] Liu MC, Oxnard GR, Klein EA, Swanton C, Seiden MV, Cummings SR, et al. Sensitive and specific multi-cancer detection and localization using methylation signatures in cell-free DNA. Ann Oncol. 2020;31:745–59. 33506766 10.1016/j.annonc.2020.02.011 PMC8274402

[10] Clarke CA, Hubbell E, Ofman JJ. Multi-cancer early detection: a new paradigm for reducing cancer-specific and all-cause mortality. Cancer Cell. 2021;39:447–8. 33606995 10.1016/j.ccell.2021.02.004

[11] Klein E, Richards D, Cohn A, Tummala M, Lapham R, Cosgrove D, et al. Clinical validation of a targeted methylation-based multi-cancer early detection test using an independent validation set. Ann Oncol. 2021;32:1167–77. 34176681 10.1016/j.annonc.2021.05.806

[12] Hackshaw A, Cohen SS, Reichert H, Kansal AR, Chung KC, Ofman JJ. Estimating the population health impact of a multi-cancer early detection genomic blood test to complement existing screening in the US and UK. Br J Cancer. 2021;125:1432–42. 34426664 10.1038/s41416-021-01498-4 PMC8575970

[13] Neal RD, Johnson P, Clarke CA, Hamilton SA, Zhang N, Kumar H, et al. Cell-free DNA–based multi-cancer early detection test in an asymptomatic screening population (NHS-Galleri): design of a pragmatic, prospective randomised controlled trial. Cancers. 2022;14:4818. 36230741 10.3390/cancers14194818 PMC9564213

[14] Hubbell E, Clarke CA, Aravanis AM, Berg CD. Modeled reductions in late-stage cancer with a multi-cancer early detection test. Cancer Epidemiol Biomark Prev. 2021;30:460–8. 10.1158/1055-9965.EPI-20-1134 33328254

[15] Bredno J, Lipson J, Venn O, Aravanis AM, Jamshidi A. Clinical correlates of circulating cell-free DNA tumor fraction. PLoS ONE. 2021;16:e0256436. 34432811 10.1371/journal.pone.0256436 PMC8386888

[16] National Cancer Registration and Analysis Service. Staging data in England. https://www.cancerdata.nhs.uk/stage_at_diagnosis. Accessed 1 November 2022.

[17] National Cancer Registration and Analysis Service. Incidence and Mortality. https://www.cancerdata.nhs.uk/incidence_and_mortality. Accessed 7 December 2022.

[18] Perme MP, Stare J, Estève J. On estimation in relative survival. Biometrics. 2012;68:113–20. 21689081 10.1111/j.1541-0420.2011.01640.x

[19] Chen X, Dong Z, Hubbell E, Kurtzman KN, Oxnard GR, Venn O, et al. Prognostic significance of blood-based multi-cancer detection in plasma cell-free DNA. Clin Cancer Res. 2021;27:4221–9. 34088722 10.1158/1078-0432.CCR-21-0417 PMC9401481

[20] Wever EM, Draisma G, Heijnsdijk EAM, de Koning HJ. How does early detection by screening affect disease progression?: Modeling estimated benefits in prostate cancer screening. Med Decis Mak. 2011;31:550–8. 10.1177/0272989X10396717 PMC4789305 21406620

[21] Office for National Statistics. National life tables, United Kingdom, 1980–1982 to 2017–2019. https://www.ons.gov.uk/peoplepopulationandcommunity/birthsdeathsandmarriages/lifeexpectancies/datasets/nationallifetablesenglandreferencetables/current. Accessed 22 November 2022.

[22] Schacht DV, Yamaguchi K, Lai J, Kulkarni K, Sennett CA, Abe H. Importance of a personal history of breast cancer as a risk factor for the development of subsequent breast cancer: results from screening breast MRI. Am J Roentgenol. 2014;202:289–92. 24450667 10.2214/AJR.13.11553

[23] National Cancer Registration and Analysis Service. National Cancer Intelligence Network, routes to diagnosis (2006–2017). https://www.gov.uk/government/statistics/routes-to-diagnosis-2006-to-2017-results/routes-to-diagnosis-2006-to-2017-results. Accessed 29 November 2022.

[24] Office for National Statistics. Mortality statistics. https://www.nomisweb.co.uk/query/construct/summary.asp?mode=construct&version=0&dataset=161#. Accessed 22 November 2022.

[25] Riggio AI, Varley KE, Welm AL. The lingering mysteries of metastatic recurrence in breast cancer. Br J Cancer. 2021;124:13–26. 33239679 10.1038/s41416-020-01161-4 PMC7782773

[26] Chabon JJ, Hamilton EG, Kurtz DM, Esfahani MS, Moding EJ, Stehr H, et al. Integrating genomic features for non-invasive early lung cancer detection. Nature. 2020;580:245–51. 32269342 10.1038/s41586-020-2140-0 PMC8230734

[27] Gale D, Heider K, Perry M, Marsico G, Ruiz-Valdepeñas A, Rundell V, et al. Residual ctDNA after treatment predicts early relapse in patients with early-stage NSCLC. J Clin Oncol. 2021;39:8517–8517. 10.1016/j.annonc.2022.02.007 PMC9067454 35306155

[28] Abbosh C, Birkbak NJ, Wilson GA, Jamal-Hanjani M, Constantin T, Salari R, et al. Phylogenetic ctDNA analysis depicts early-stage lung cancer evolution. Nature. 2017;545:446–51. 28445469 10.1038/nature22364 PMC5812436

[29] Bredno J, Venn O, Chen X, Freese P, Ofman JJ. Circulating Tumor DNA allele fraction: a candidate biological signal for multicancer early detection tests to assess the clinical significance of cancers. Am J Pathol. 2022;192:1368–78. 35948080 10.1016/j.ajpath.2022.07.007

[30] Avanzini S, Kurtz DM, Chabon JJ, Moding EJ, Hori SS, Gambhir SS, et al. A mathematical model of ctDNA shedding predicts tumor detection size. Sci Adv. 2020;6:eabc4308. 10.1126/sciadv.abc4308 PMC7732186 33310847

[31] Croswell JM, Kramer BS, Kreimer AR, Prorok PC, Xu JL, Baker SG, et al. Cumulative incidence of false-positive results in repeated, multimodal cancer screening. Ann Fam Med. 2009;7:212–22. 19433838 10.1370/afm.942 PMC2682972

[32] Welch HG, Black WC. Overdiagnosis of cancer. J Natl Cancer Inst. 2010;102:605–13. 20413742 10.1093/jnci/djq099

[33] Hubbell E, Clarke C. Detecting cancer when it can be cured: The potential for cure across all stageable cancers. Cancer Res. 2022;82:2239.35395674

[34] National Cancer Registration and Analysis Service. 10-year survival by stage for the East of England. http://www.ncin.org.uk/about_ncin/10yearsurvival. Accessed 22 November 2022.

[35] Hanagiri T, Takenaka M, Oka S, Shigematsu Y, Nagata Y, Shimokawa H, et al. Results of a surgical resection for patients with Stage IV non–small-cell lung cancer. Clin Lung Cancer. 2012;13:220–4. 22138036 10.1016/j.cllc.2011.05.006

[36] Liu S, Zhou F, Liu Z, Xiong A, Jia Y, Zhao S, et al. Predictive and prognostic significance of M descriptors of the 8th TNM classification for advanced NSCLC patients treated with immune checkpoint inhibitors. Transl Lung Cancer Res. 2020;9:1053–66. 32953484 10.21037/tlcr-19-396 PMC7481592

[37] David EA, Clark JM, Cooke DT, Melnikow J, Kelly K, Canter RJ. The role of thoracic surgery in the therapeutic management of metastatic non–small cell lung cancer. J Thorac Oncol. 2017;12:1636–45. 28843357 10.1016/j.jtho.2017.08.008 PMC5690551

[38] Lo Dico R, Faron M, Yonemura Y, Glehen O, Pocard M, Sardi A, et al. Combined liver resection and cytoreductive surgery with HIPEC for metastatic colorectal cancer: results of a worldwide analysis of 565 patients from the Peritoneal Surface Oncology Group International (PSOGI). Eur J Surg Oncol. 2021;47:89–100. 32943276 10.1016/j.ejso.2020.07.038

[39] Zou X, You R, Liu H, He YX, Xie GF, Xie ZH, et al. Establishment and validation of M1 stage subdivisions for de novo metastatic nasopharyngeal carcinoma to better predict prognosis and guide treatment. Eur J Cancer. 2017;77:117–26. 28391025 10.1016/j.ejca.2017.02.029

[40] Pagani O, Senkus E, Wood W, Colleoni M, Cufer T, Kyriakides S, et al. International guidelines for management of metastatic breast cancer: Can metastatic breast cancer be cured? J Natl Cancer Inst. 2010;102:456–63. 20220104 10.1093/jnci/djq029 PMC3298957

[41] Elkin EB, Hudis C, Begg CB, Schrag D. The effect of changes in tumor size on breast carcinoma survival in the U.S.: 1975–1999. Cancer. 2005;104:1149–57. 16088887 10.1002/cncr.21285

[42] McPhail S, Swann R, Johnson SA, Barclay ME, Abd Elkader H, Alvi R, et al. Risk factors and prognostic implications of diagnosis of cancer within 30 days after an emergency hospital admission (emergency presentation): an International Cancer Benchmarking Partnership (ICBP) population-based study. Lancet Oncol. 2022;23:587–600. 35397210 10.1016/S1470-2045(22)00127-9 PMC9046095

[43] Carreira H, Williams R, Dempsey H, Stanway S, Smeeth L, Bhaskaran K. Quality of life and mental health in breast cancer survivors compared with non-cancer controls: a study of patient-reported outcomes in the United Kingdom. J Cancer Surviv. 2021;15:564–75. 33089480 10.1007/s11764-020-00950-3 PMC8272697

