# Correction: Modelled mortality benefits of multi-cancer early detection screening in England

**DOI:** 10.1038/s41416-024-02836-y

**Published:** 2024-11-18

**Authors:** Peter Sasieni, Rebecca Smittenaar, Earl Hubbell, John Broggio, Richard D. Neal, Charles Swanton

**Affiliations:** 1 Comprehensive Cancer Centre, King’s College London, Guy’s Campus, Great Maze Pond, London, SE1 1UL UK; 2 GRAIL Bio UK Ltd, a subsidiary of GRAIL, LLC, London, WC1V 7HP UK; 3 GRAIL, LLC, Menlo Park, CA 94025 USA; 4 NHS Digital, 7 and 8 Wellington Place, Leeds, West Yorkshire LS1 4AP UK; 5 Department of Health and Community Sciences, Faculty of Health and Life Sciences, University of Exeter, Exeter, UK; 6 Cancer Research UK Lung Cancer Centre of Excellence, University College London Cancer Institute, London, WC1E 6DD UK; 7 Cancer Evolution and Genome Instability Laboratory, Francis Crick Institute, London, NW1 1AT UK

**Keywords:** Cancer epidemiology, Cancer screening

Correction to: *British Journal of Cancer* 10.1038/s41416-023-02243-9, published online 25 April 2023

Following publication of the article, the authors noticed errors in the model code on re-examination. Data has been corrected in Tables 1, 2, 3 and 4 in the main article; Tables S4A and S4B in the supplementary material have also been updated.

**Corrected Table 1**

**Table 1:** Late-Stage Incidence Reduction in the Open Cohort Scenario.

		Dwell Time Scenario		
	No Screening	Slow	Medium	Fast
**Prevalent Screening Round**				
**Basic Performance (Including Future Year Cancers Found Now)**				
Found Via Usual Care and MCED (%)	NA	3768 (100)	2847 (100)	2150 (100)
Found Via Usual Care (%)	NA	1003 (27)	1056 (37)	1151 (54)
MCED Detected (%)	NA	2765 (73)	1791 (63)	999 (46)
**Late-Stage Reduction (Including Future Year Cancers Found Now)**				
Late-Stage Diagnosis With Usual Care (%)	NA	2401 (64)	1688 (59)	1128 (52)
Late-Stage Diagnosis With MCED (%)	NA	799 (21)	690 (24)	573 (27)
Reduction in Late-Stage Diagnosis With MCED (%)	NA	1602 (67)	999 (59)	555 (49)
**Incident Screening Round**				
**Basic Performance (Steady State)**				
Found Via Usual Care and MCED (%)	1192 (100)	1192 (100)	1192 (100)	1192 (100)
Found Via Usual Care (%)	1192 (100)	727 (61)	764 (64)	833 (70)
MCED Detected (%)	NA	465 (39)	428 (36)	359 (30)
**Late-Stage Incidence Reduction** (Steady State)				
Late-Stage Diagnosis With Usual Care (%)	506 (42)	506 (42)	506 (42)	506 (42)
Late-Stage Diagnosis With MCED (%)	NA	257 (22)	276 (23)	305 (26)
Reduction in Late-Stage Diagnosis With MCED (%)	NA	248 (49)	230 (45)	201 (40)

For the ‘open cohort’ modelled scenario, the impact of multi-cancer early detection (MCED) screening on the incidence rate of cancers diagnosed at a late stage (III and IV) is shown for each dwell time scenario and for prevalent and incident screening rounds. These results are directly comparable to estimates produced for introducing a screening programme based on an MCED test, with sensitivity as estimated in a case–control study (11), to the US population (14). All results are incidence rates per 100,000 persons screened. Prevalent rounds of screening were most susceptible to dwell time assumptions. This is reflected in the mortality rates without MCED screening because there are different numbers of cancers available to be diagnosed through usual care, depending on assumptions regarding the speed of dwell times. This is in contrast with the incident rounds of screening, in which the previous prevalent rounds ‘level-set’ the numbers of cancers available for diagnosis. As the model is focused on screening referral performance, we did not explicitly model the diagnostic resolution process, which may be extended due to work-up at multiple potential cancer signal origins, or terminated after a single diagnostic work-up, with the risk of missing a cancer. Instead, we made the simplifying assumption that diagnostic resolution occurs shortly after detection. Another simplifying assumption was that ‘usual care’ refers to the standard practice in terms of screening, primary care referral, diagnostic work-up and treatment resulting in the incidence-by-stage and survival-by-stage data observed in the period 2013–2018 in England. This assumption was to incorporate non-adherence to screening, diagnostic and treatment guidance, and limited access to healthcare. The denominator for the percentage calculations corresponds to all staged and unstageable cancers. Stageable cancers with missing stage information were excluded from the analysis. Data are to the nearest whole number; therefore, breakdowns of total numbers may not always sum perfectly.

**Corrected Table 2**

**Table 2:** Five-Year Mortality Rate Reduction in the Open Cohort Scenario.

	No Screening	Hazard Ratio = 1			Hazard Ratio = 2			Hazard Ratio = 3		
		Slow	Medium	Fast	Slow	Medium	Fast	Slow	Medium	Fast
**Prevalent Screening Round (Including Future Year Cancers Found Now)**										
Cancer Mortality Rate With Usual Care	NA	1954	1375	964	2007	1405	975	2036	1420	981
Cancer Mortality Rate With MCED	NA	1268	941	714	1386	1010	750	1451	1048	770
Reduction in Cancer Mortality Rate With MCED, (%)	NA	686 (35)	434 (32)	250 (26)	621 (31)	395 (28)	225 (23)	585 (29)	372 (26)	211 (22)
**Incident Screening Round (Steady State)**										
Cancer Mortality Rate With Usual Care	442	442	442	442	442	442	442	442	442	442
Cancer Mortality Rate With MCED	NA	335	343	355	346	352	363	352	358	368
Reduction in Cancer Mortality Rate With MCED (%)	NA	107 (24)	99 (22)	87 (20)	96 (22)	90 (20)	79 (18)	90 (20)	84 (19)	74 (17)

For the ‘open cohort’ modelled scenario, the impact of screening with a multi-cancer early detection (MCED) test with sensitivity as estimated in a case–control study (11) on the five-year cancer mortality rate is shown per 100,000 persons screened. Results are displayed for each dwell time scenario and for differential survival hazard ratios (HRs) for cell-free DNA^+^ (cfDNA^+^) cancers versus cfDNA^–^ cancers. HR = 1 corresponds to no increased risk of mortality with a cfDNA^+^ cancer. Prevalent rounds of screening are most susceptible to dwell time assumptions, as reflected in the late-stage incidence reduction results in Table 1. There is additional complexity in that different HRs contribute to different mortality rates between scenarios, even without MCED screening, because cfDNA^+^ cancers are present irrespective of whether or not they are intercepted. The data should be interpreted as follows: for the fast dwell time scenario, there were 1717 cancers diagnosed in the prevalent round of screening (Table 1), and assuming HR = 1, there were 770 deaths from cancers diagnosed via usual care, and 570 deaths from cancers diagnosed when MCED screening was added to usual care. This corresponds to a difference in the five-year cancer mortality rate of 200 per 100,000 persons screened (26%) due to the addition of MCED screening to usual care. Data are to the nearest whole number; therefore, breakdowns of total numbers may not always sum perfectly.

**Corrected Table 3**

**Table 3:** Late-Stage Incidence and Five-Year Mortality Rate Reductions by Cancer Type.

	Current Incidence Rate	Late-Stage Diagnosis With Usual Care	Late-Stage Diagnosis With MCED	Reduction in Late-Stage Diagnosis With MCED (%)	Cancer Mortality Rate With Usual Care	Cancer Mortality Rate With MCED	Reduction in Mortality With MCED (%)
Colon/ Rectum	129	71	24	47 (66)	45	21	25 (56)
Lung	155	113	49	64 (57)	126	108	18 (14)
Ovary	26	17	5	12 (71)	14	6	7 (50)
Head and Neck	42	28	7	22 (79)	16	8	8 (50)
Breast	176	23	14	9 (39)	14	10	4 (29)
Liver/ Bile Duct	22	15	4	11 (73)	18	14	4 (22)
Pancreas	32	25	11	14 (56)	30	27	3 (10)
Oesophagus	31	23	12	10 (43)	24	22	2 (8)
Lymphoma	50	34	17	17 (50)	14	11	3 (21)
Prostate	195	81	77	3 (4)	16	14	2 (12)
Bladder	29	8	6	1 (12)	12	10	2 (17)
Cervix	5	2	0	1 (50)	2	1	1 (50)
Kidney	37	16	14	2 (12)	11	10	1 (9)
Sarcoma	9	4	2	2 (50)	4	3	1 (25)
Stomach	18	13	8	5 (38)	14	13	1 (7)
Uterus	34	6	4	2 (33)	6	5	1 (17)
Anus	5	3	1	2 (67)	1	1	0 (0)
Gallbladder	7	4	4	1 (25)	5	5	0 (0)
Melanoma	51	5	5	0 (0)	4	4	0 (0)
Thyroid	8	4	4	0 (0)	1	1	0 (0)
Urothelial Tract	6	4	4	0 (0)	3	3	0 (0)

For adults aged 50–79 years, for each cancer type, the table shows: crude incidence rates (per 100,000 persons); incidence rates for late-stage (III & IV) cancer diagnoses with usual care, and with multi-cancer early detection (MCED) screening (with sensitivity as estimated in a case–control study [11]) added to usual care; incidence rates of cancers shifted early by MCED screening; five-year mortality rate reductions (per 100,000 persons screened) for usual care, and with MCED screening added to usual care. For mortality rates, we used a survival hazard ratio (HR) of three for cell-free DNA^+^ cancers versus cfDNA^–^ cancers, to provide the most conservative estimate of benefit from MCED screening. Data are to the nearest whole number; therefore, breakdowns of total numbers may not always sum perfectly. The table has been sorted by absolute reduction in mortality with MCED screening.

**Corrected Table 4**

**Table 4:** Late-Stage Incidence and Five-Year Mortality Rate Reductions in a National Screening Programme Scenario.

Late-Stage Incidence and Five-Year Mortality Rate Reductions, National Screening Programme (Per 100,000 Persons Entering Screening Programme)			
Dwell Time Scenario	Slow	Medium	Fast
**Basic Performance**			
Found Via Usual Care (%)	17,412 (60)	18,313 (64)	19,947 (70)
Found Via MCED (%)	11,614 (40)	10,515 (36)	8706 (30)
**Late-Stage Incidence Reduction**			
Late-Stage Diagnosis With MCED (%)	6249 (24)	6666 (25)	7327 (28)
Reduction in Late-Stage Diagnosis with MCED (%)	6217 (50)	5658 (46)	4865 (40)
**Five-Year Cancer Mortality Rate**			
**HR cfDNA = 1**			
Cancer Mortality Rate With Usual Care (%)	8123 (28)	8265 (29)	8517 (30)
Reduction in Cancer Mortality Rate With MCED (%)	2664 (25)	2437 (23)	2115 (20)
**HR cfDNA = 2**			
Cancer Mortality Rate With Usual Care (%)	8393 (29)	8508 (30)	8729 (30)
Reduction in Cancer Mortality Rate with MCED (%)	2404 (22)	2199 (21)	1905 (18)
**HR cfDNA = 3**			
Cancer Mortality With Rate Usual Care (%)	8544 (29)	8645 (30)	8848 (31)
Reduction in Cancer Mortality Rate With MCED (%)	2257 (21)	2064 (19)	1787 (17)

This table shows cumulative benefits of a national screening programme based on a multi-cancer early detection (MCED) test, with sensitivity as estimated in a case–control study (11), starting from 50 years of age with annual screening until 79 years of age for three dwell time scenarios; mortality rate reduction outcomes are presented for three cfDNA hazard ratio (HR) scenarios. Data are to the nearest whole number; therefore, breakdowns of total numbers may not always sum perfectly.

The correction does not have any effect on the results or conclusions of the paper.

The original article has been corrected.

## Supplementary information

Supplementary Information_Revised

