# Supplementary material for: Modelled mortality benefits of multi-cancer early detection screening in England
Source: Br J Cancer. 2023 Apr 25;129(1):72–80. doi: 10.1038/s41416-023-02243-9 (PMC10307803; doi:10.1038/s41416-023-02243-9)
Supplement: Supplementary file 1 — Supplementary Information [file 41416_2023_2243_MOESM1_ESM.pdf]

Supplementary Information

Table S1. Definitions of Invasive Cancer Types.

Cancer types were defined according to the International Classification of Diseases for Oncology, Third Edition, First Revision (ICD-O-3.1). Classifications were mapped to the performance of one multi-cancer early detection (MCED) test (Galleri®) and generally involve broad histologic categorizations (e.g., sarcoma, lymphoma, melanoma) excluded from categorizations of solid organ sites. The 'other' cancer category consists of both staged and unstaged rarer cancers.

Cancer Type	ICD-03 Site and Histology Code Definition
**Anus**	All C210–C218 excluding histology 8140, 8710–8931, 9040–9055, 9120–9342, 9580–9992; C180–C199, C209, C260 with histology 8070–8071
**Bladder**	All C670–C679 excluding histology 8710–8931, 9040–9055, 9120–9342, 9580–9992
**Breast**	All C500–C506, C508, C509 excluding histology 8710–8931, 9040–9055, 9120–9342, 9580–9992
**Cervix**	All C530, C531, C538, C539 excluding histology 8710–8931, 9040–9055, 9120–9342, 9580–9992
**Colon/Rectum**	All C180–C199, C209, C260 excluding histology 8710–8931, 9040–9055, 9120–9342, 9580–9992; C210–218 with histology 8140
**Gallbladder**	All C239, C240–249 excluding histology 8710–8931, 9040–9055, 9120–9342, 9580–9992
**Head and Neck**	All C000–C148, C300–C329 excluding histology 8710–8931, 9040–9055, 9120–9342, 9580–9992
**Kidney**	C649 excluding histology 8120, 8122, 8130, 8710–8931, 9040–9055,

	9120–9342, 9580–9992
**Liver/Bile Duct**	All C220–C221 excluding histology 8710–8931, 9040–9055, 9120–9342, 9580–9992
**Lung**	All C340–C349 excluding histology 8710–8931, 9040–9055, 9120–9342, 9580–9992
**Lymphoid Leukaemia**	All histology 9712, 9728, 9729, 9811–9820, 9823, 9827, 9831–9837, 9940, 9948
**Lymphoma**	All histology 9590–9597, 9650–9667, 9670–9671, 9673, 9675, 9678–9680, 9684, 9687–9691, 9695, 9698–9702, 9705, 9708–9709, 9714–9719, 9724–9727, 9735, 9737–9738, 9760–9761, 9764, 9826, 9838, 9970–9971
**Melanoma**	All histology 8720–8790
**Myeloid Neoplasm**	All histology 9740–9742, 9751, 9801–9809, 9840, 9860–9876, 9891–9898, 9910–9911, 9920, 9930–9939, 9941–9946, 9963–9964, 9966, 9975
**Plasma Cell Neoplasm**	All histology 9731–9734, 9762
**Oesophagus**	All C150–C159 excluding histology 8710–8931, 9040–9055, 9120–9342, 9580–9992
**Ovary**	All C569, C570, C481, C482, C488 excluding histology 8710–8931, 9040–9055, 9120–9342, 9580–9992
**Pancreas**	All C250–C259 excluding histology 8710–8931, 9040–9055, 9120–9342, 9580–9992
**Prostate**	All C619 excluding 8710–8931, 9040–9055, 9120–9342, 9580–9992

**Sarcoma**	All histology including 8710, 8711, 8800–8931, 9040–9044, 9120–9342, 9580, 9581
**Stomach**	All C160–C169 excluding histology 8710–8931, 9040–9055, 9120–9342, 9580–9992
**Thyroid**	All C739 excluding histology 8710–8931, 9040–9055, 9120–9342, 9580–9992
**Urothelial Tract**	All C659, C669, C680 excluding histology 8710–8931, 9040–9055, 9120–9342, 9580–9992; and all C649 with histology 8120, 8122, 8130
**Uterus**	C540–C543, C548–C549, C559 excluding histology 8710–8931, 9040–9055, 9120–9342, 9580–9992
**Other**	All other sites not categorised above

Table S2. Incidence Rate per 100,000 Persons by Cancer Type and Stage.

Stageable cancers with missing stage data have been excluded from further analysis. This is a conservative assumption that underestimates the benefit of MCED screening: within this group, there may be cancers for which late-stage incidence reduction could occur. Cubic spline-based interpolation was used to create one-year age bands, with a smooth estimate per year of age.

	**Stage at Diagnosis**					
**Cancer**	**I**	**II**	**III**	**IV**	**Unknown/ Missing**	**Unstageable**
**Anus**	0.66	1.21	1.85	0.45	0.93	
**Bladder**	12.80	7.08	2.36	4.48	3.62	
**Breast**	81.92	60.79	13.40	8.05	12.37	
**Cervix**	1.26	1.10	0.42	0.72	1.28	
**Colon/Rectum**	23.72	30.58	36.80	29.82	10.80	
**Gallbladder**	0.46	1.06	0.91	2.28	2.09	

**Head and Neck**	7.61	4.55	5.08	19.66	5.67	
**Kidney**	15.36	2.73	6.51	7.42	5.33	
**Liver/Bile Duct**	1.49	1.70	1.51	5.13	12.52	
**Lung**	27.88	11.89	31.73	75.84	10.97	
**Lymphoid Leukaemia**						16.31
**Lymphoma**	7.84	5.74	8.38	20.04	9.22	
**Melanoma**	31.24	9.37	3.36	1.37	6.46	
**Myeloid Neoplasm**						13.71
**Oesophagus**	2.94	3.89	9.40	10.36	4.34	
**Ovary**	6.69	1.48	9.07	5.18	4.16	
**Pancreas**	1.70	3.93	3.17	16.85	6.93	
**Plasma Cell Neoplasm**						19.50
**Prostate**	68.50	39.09	43.59	31.75	17.45	
**Sarcoma**	1.30	1.04	1.03	0.94	5.43	
**Stomach**	1.81	2.51	3.58	7.49	3.55	
**Thyroid**	2.30	0.94	1.16	1.37	2.73	
**Urothelial Tract**	1.08	0.69	1.21	1.75	1.34	
**Uterus**	24.34	2.15	3.47	2.11	2.57	
**Other**	5.02	2.25	4.09	4.47	60.92	

Table S3A. Survival by Cancer Type and Stage.

We carried out survival analyses based on persons for all cancers except sex-specific cancers (prostate, uterus, cervix, and ovary).

**Cancer Type**	**Five-Year Net Survival (%)**				
	**I**	**II**	**III**	**IV**	**Unstageable**
**Anus**	92.19	83.15	68.96	17.49	
**Bladder**	83.24	50.41	45.21	13.09	
**Breast**	99.75	93.21	76.39	28.38	
**Cervix**	94.91	69.08	38.52	15.93	

**Colon/Rectum**	95.49	86.34	70.15	12.33	
**Gallbladder**	54.48	39.99	19.70	2.68	
**Head and Neck**	89.24	74.90	67.19	48.52	
**Kidney**	92.29	86.36	77.32	14.65	
**Liver/ Bile Duct**	47.48	35.71	11.91	2.53	
**Lung**	58.93	36.79	13.70	2.85	
**Lymphoid Leukaemia**					84.59
**Lymphoma**	85.58	80.73	71.40	65.38	
**Melanoma**	99.90	80.87	69.15	29.38	
**Myeloid Neoplasm**					30.84
**Oesophagus**	58.93	36.97	19.92	2.91	
**Ovary**	93.51	71.84	29.24	16.52	
**Pancreas**	31.61	18.63	5.71	1.56	
**Plasma Cell Neoplasm**					57.88
**Prostate**	99.90	99.90	99.43	53.47	
**Sarcoma**	81.22	68.79	50.72	6.80	
**Stomach**	70.79	38.50	26.24	3.69	
**Thyroid**	99.90	99.78	96.88	53.27	
**Urothelial Tract**	81.66	68.91	52.02	11.51	
**Uterus**	94.21	78.41	51.71	18.81	
**Other**	78.22	61.72	40.71	19.84	47.01

Table S3B. Reliability of Cancer Survival Estimates.

For some combinations of cancer site, stage, and age band, there were no events and therefore survival could not be calculated. In those cases, we imputed data by assuming the survival of the

nearest relevant older age band. This is a conservative assumption as survival in the older age group is typically worse. Net survival estimates were considered potentially unreliable when one of the following criteria were met: standard error (SE) > 0.2; net survival estimate > 1 (e.g., in breast cancer); number of patients < 5; and/or difference between upper and lower confidence intervals (CIs) > 20 (as shown in the table below). This is because the calculation is based on small numbers of events due to stratification by cancer type, stage and five-year age band. For staged cancers, there were 24 estimates of survival, and for unstageable cancers there were six estimates of survival. This impacted low-incidence cancers, including anus, gallbladder, cervix and urothelial tract, in particular; in addition, prostate cancer, which has high early-stage survival, has a proportionally large number of potentially unreliable estimates because there are few deaths ('events'). Survival estimates for cancers with a high proportion of potentially unreliable estimates should be interpreted with caution. Linear interpolation was used to create 1-year age bands, with a smooth estimate per year of age.

**Potentially Unreliable Five-Year Net Survival Estimates**							
		**Five-year age bands**					
**Cancer Type**	**Stage**	**50–54**	**55–59**	**60–64**	**65–69**	**70–74**	**75–79**
**Anus**	**I**	1	1	0	1	1	1
	**II**	1	0	0	0	1	1
	**III**	0	0	0	0	1	1
	**IV**	1	1	1	1	1	1
**Bladder**	**I**	0	0	0	0	0	0
	**II**	0	0	0	0	0	0
	**III**	1	1	0	0	0	0
	**IV**	0	0	0	0	0	0
**Breast**	**I**	0	0	0	1	1	1
	**II**	0	0	0	0	0	0

	**III**	0	0	0	0	0	0
	**IV**	0	0	0	0	0	0
**Cervix**	**I**	0	0	1	1	1	1
	**II**	0	1	1	0	1	1
	**III**	1	1	1	1	1	1
	**IV**	1	1	0	1	1	1
**Colon/Rectum**	**I**	0	0	0	0	0	0
	**II**	0	0	0	0	0	0
	**III**	0	0	0	0	0	0
	**IV**	0	0	0	0	0	0
**Gallbladder**	**I**	1	1	1	1	1	1
	**II**	1	1	1	0	1	1
	**III**	1	1	1	1	0	1
	**IV**	1	0	1	1	1	1
**Head and Neck**	**I**	0	0	0	0	0	0
	**II**	0	0	0	0	0	0
	**III**	0	0	0	0	0	0
	**IV**	0	0	0	0	0	0
**Kidney**	**I**	0	0	0	0	0	0
	**II**	0	0	0	0	0	0
	**III**	0	0	0	0	0	0
	**IV**	0	0	0	0	0	0
**Liver/Bile Duct**	**I**	1	1	1	0	1	1

	**II**	1	1	1	0	1	1
	**III**	1	1	1	0	1	1
	**IV**	1	1	1	1	1	1
**Lung**	**I**	0	0	0	0	0	0
	**II**	0	0	0	0	0	0
	**III**	0	0	0	0	0	0
	**IV**	0	0	0	0	0	0
**Lymphoid Leukaemia**	**Unstageable**	0	0	0	0	0	0
**Lymphoma**	**I**	0	0	0	0	0	0
	**II**	0	0	0	0	0	0
	**III**	0	0	0	0	0	0
	**IV**	0	0	0	0	0	0
**Melanoma**	**I**	0	1	1	1	1	1
	**II**	0	0	0	0	0	0
	**III**	0	0	0	0	0	0
	**IV**	1	0	0	0	0	1
**Myeloid Neoplasm**	**Unstageable**	0	0	0	0	0	0
**Oesophagus**	**I**	1	0	0	0	0	0
	**II**	1	0	0	0	0	0
	**III**	0	0	0	0	0	0
	**IV**	1	0	0	0	0	1
**Ovary**	**I**	0	0	0	0	0	0
	**II**	1	0	0	1	1	1

	**III**	0	0	0	0	0	0
	**IV**	0	0	0	0	0	0
**Pancreas**	**I**	1	1	0	0	0	0
	**II**	0	0	0	0	0	0
	**III**	1	1	1	1	1	1
	**IV**	0	0	0	0	0	1
**Plasma Cell Neoplasm**	**Unstageable**	0	0	0	0	0	0
**Prostate**	**I**	1	1	1	1	1	1
	**II**	1	1	1	1	1	1
	**III**	0	0	0	0	0	1
	**IV**	0	0	0	0	0	0
**Sarcoma**	**I**	0	0	0	1	1	1
	**II**	1	1	1	1	1	1
	**III**	1	1	1	1	1	1
	**IV**	1	1	1	1	1	1
**Stomach**	**I**	1	1	1	0	0	0
	**II**	0	0	0	0	0	0
	**III**	0	0	0	0	0	0
	**IV**	0	0	0	0	0	0
**Thyroid**	**I**	1	0	1	1	1	1
	**II**	1	1	1	1	1	1
	**III**	1	0	1	1	1	1
	**IV**	0	0	1	0	0	1

**Urothelial Tract**	**I**	1	1	0	1	1	1
	**II**	1	1	1	1	1	1
	**III**	1	1	1	1	1	1
	**IV**	1	1	1	0	0	1
**Uterus**	**I**	0	0	0	0	0	0
	**II**	0	0	0	0	0	1
	**III**	0	0	0	0	0	0
	**IV**	1	0	0	0	0	0
**Other**	**I**	0	0	0	0	0	0
	**II**	0	0	0	0	0	0
	**III**	0	0	0	0	0	0
	**IV**	0	0	0	0	0	0
	**Unstageable**	0	0	0	0	0	0

Analysis S4. Non-Sequential Stage Progression Model.

Introduction

To consider the implications for the benefit of screening based on a MCED test among cancers with non-sequential stage progression, we developed a mixture model whereby different proportions of cancers that are currently clinically diagnosed at stage IV under usual care are assumed to have either (A) a short stage I (of six months in length), or (B) no previous stages. These models represent a range of different biological scenarios including cancers metastasizing directly from a preclinical or stage I cancer, as well as metastatic subclones originating from local tumours; the biology of these cancers not only leads to distant metastases before local spread, but also accelerate progression from the earliest stage of disease.

Methods

We implemented model A by setting the dwell time in stage I to be exponentially distributed around six months, and the dwell times in stages II and III to be 0.0001 with slip rate = 1. In this model, there was no stage II or III at which the cancers can be intercepted, and there was only benefit from MCED screening if the cancer was intercepted in stage I. Model B was implemented by setting the dwell time in stages I, II and III to 0.0001, with slip rate = 1. MCED screening cannot confer any benefit to cancers with this biology, as there are no prior stages in which cancers can be intercepted. This non-sequential progression was restricted to cancers currently clinically diagnosed at stage IV.

We applied these models to the 11 of the 12 pre-specified cancers with Tumour-Node-Metastasis (TNM) staging systems (anus, bladder, colon/rectum, head and neck, liver/bile duct, lung, lymphoma, oesophagus, ovary, pancreas and stomach cancers). Based on studies in lung cancer, high levels of cfDNA-shedding have been linked to mechanisms by which cancers metastasize directly from a preclinical stage or stage I (25). Despite limited research on the behaviours of high-cfDNA-shedding tumours in other cancer types, for the purposes of modelling this worst-case scenario in terms of high cfDNA levels, we included these high-sensitivity/high-cfDNA-shedding cancer types in the non-sequential stage progression scenario. For all other cancers, stage progression was assumed to be sequential throughout.

These models were mixed with the main sequential stage progression model, with three different proportions of non-sequential stage progression for the 11 pre-specified cancers: 0.1, 0.25, and 0.5.

In these models, we used the medium dwell time assumption with hazard ratio (HR) = 3 for cfDNA-detectable cancers. We used the national screening programme framework to better understand the long-term benefits of an MCED screening programme with these various proportions of cancers with non-sequential stage progression, as the open cohort may be unduly influenced by the prevalent round of screening.

Results

Results are displayed in Tables 4A and S4B. The number of cancers available to be found via usual care (i.e., without MCED screening) increased with the proportion of cancers that have non-sequential progression. Decreasing the amount of time spent in stage I brought forward in time all cancers that would have otherwise been diagnosed clinically in subsequent years.

Similarly, with increased proportions of cancers with non-sequential progression, the diagnostic yield via MCED screening was smaller. This reflects limited or no opportunities for MCED detection among these cancers, for which the MCED test typically has high sensitivity, at earlier stages. When these cancers are diagnosed via MCED screening at stage IV, no mortality benefit is conferred for these tumours. For diagnosis via MCED screening, the number of cancers diagnosed at a late stage increased as the proportion of non-sequential progression cancers increased. This is because when cancers spend no time in any stages prior to stage IV, intercepting these cancers earlier and reducing the amount of late-stage diagnoses is not possible. In the most adverse scenario (in which 50% of cancers have non-sequential progression, with no time spent in stages prior to stage IV), 33% of cancers were estimated to be diagnosed late via usual care and MCED screening. The cancer mortality rate in the absence of MCED screening was highest (33%) in the scenario with the most adverse cancer biology, as more cancers with the most rapidly lethal biologies will be diagnosed during the national screening programme period. However, cancer mortality was 31% in the absence of MCED in the national screening programme scenario with a 'fast' dwell time and HR = 3 for $cfDNA^+$ tumours (Table 4); this suggests that the most adverse scenario may be too pessimistic. Again, in the most adverse scenario, the reduction in cancer mortality was 13%, which is 6% lower than the cancer mortality reduction in the sequential progression model with a medium dwell time and HR = 3 (Table 4).

Table S4A. Model A: Benefits of Multi-Cancer Early Detection (MCED) Screening in a Non-Sequential Stage Progression Framework, with Six Months in Stage I.

National screening programme, medium dwell time scenario, HR = 3 for cfDNA status. The total number of cancers found via usual care and via screening with an MCED test with sensitivity as estimated in a case–control study (11) when added to usual care differed between each of the non-sequential stage progression scenarios. This reflects the fact that non-sequential stage progression reduces the effective time spent in the pre-clinical state, which affects both the prevalent round at the start of the programme, and the last round of the programme.

	Proportion of Cancers with Non-Sequential Progression

	0.1	0.25	0.5
**Basic Performance**			
**Found Via Usual Care (%)**	18,528 (64)	18,850 (65)	19,388 (67)
**Found Via MCED (%)**	10,288 (36)	9947 (35)	9380 (33)
**Late-Stage Incidence Reduction**			
**Late-Stage Diagnosis With MCED (%)**	6890 (26)	7226 (28)	7786 (30)
**Reduction in Late-Stage Diagnosis with MCED (%)**	5422 (44)	5068 (41)	4478 (37)
**Cancer Mortality Rate in Five Years**			
**Cancer Mortality with Usual Care (%)**	8733 (30)	8865 (31)	9086 (32)
**Reduction in Cancer Mortality Rate with MCED (%)**	1969 (18)	1827 (17)	1589 (15)

Table S4B. Model B: Benefits of Multi-Cancer Early Detection (MCED) Screening in a Non-Sequential Stage Progression Model for any Cancer Diagnosed at Stage IV, with No Earlier Stages.

National screening programme, medium dwell time scenario, HR = 3 for cfDNA status. The total number of cancers found via usual care and via screening with an MCED test when added to usual care differed between the non-sequential stage progression scenarios. As in Model A, this reflects the fact that non-sequential stage progression reduces the effective time spent in a preclinical state, which affects both the prevalent round at the start of the programme, and the last round of the programme.

	Proportion of Cancers with Non-Sequential Stage Progression		
	**0.1**	**0.25**	**0.5**
**Basic Performance**			
**Found Via Usual Care (%)**	**18,587** (65)	18,997 (66)	19,680 (68)
**Found Via MCED (%)**	**10,229** (35)	9800 (34)	9085 (32)
**Late-Stage Incidence Reduction**			
**Late-Stage Diagnosis With MCED (%)**	**6974** (27)	7435 (28)	8204 (31)
**Reduction in Late-Stage Diagnosis With MCED (%)**	**5338** (43)	4858 (40)	4058 (33)
**Cancer Mortality Rate in Five Years**			
**Cancer Mortality Rate With Usual Care (%)**	**8767** (30)	8949 (31)	9254 (32)
**Reduction in Cancer Mortality Rate With MCED (%)**	**1935** (18)	1742 (16)	1419 (13)

Discussion

By its nature, there is a lot of uncertainty around the natural history of cancer prior to clinical diagnosis. Here, we modelled possible scenarios to estimate the impact of non-sequential stage progression on the benefits of MCED screening for a group of 11 pre-specified cancers. More flexible models permitting rapid progression to metastatic disease, with limited or no opportunities for detection at prior stages, generally reduced the predicted effectiveness of screening. However, screen detection at stage I for cancers currently diagnosed in usual care at stage IV, as well as earlier detection of cancers currently diagnosed in usual care at intermediate stages, may still result in considerable benefit from screening. Whether this level of benefit, or indeed the benefit gained under the most favourable set of assumptions, is sufficient to outweigh

the harms associated with false positives remains to be seen. The resource implications of a MCED screening programme are also currently unknown. We will gain insight into these issues, and more fundamental issues such as the natural history of many of these cancers, in the NHS-Galleri trial. It will be important to revisit the structural assumptions related to natural history when the results of this trial and others become available, and to triangulate the evidence from fundamental studies into the evolution of cancers.

Table S5. Galleri® Test Sensitivity.

Sensitivity of a commercially available MCED test (Galleri®) was reported by Klein and colleagues (2020) and values have been adjusted by weighted isotonic regression so that sensitivity increases by stage. For the purposes of the model, lymphoid leukaemia, myeloid neoplasm, and plasma cell neoplasm were considered to be unstageable. These cancers have a single sensitivity estimate based on data from Klein and colleagues (2020), and contribute to results regarding the numbers of cancers detected by screening (basic performance), but not late-stage incidence or mortality reduction outcomes.

Cancer Type	Sensitivity by Stage				
	Stage I	Stage II	Stage III	Stage IV	Single Sensitivity
**Anus**	0.25	0.75	1	1	
**Bladder**	0.1765	0.1765	0.75	1	
**Breast**	0.0264	0.4751	0.8545	0.9090	
**Cervix**	0.5833	1	1	1	
**Colon/Rectum**	0.4333	0.85	0.8788	0.9531	
**Gallbladder**	0	0.3333	0.75	1	
**Head and Neck**	0.6316	0.8236	0.8421	0.96	
**Kidney**	0.0492	0.1875	0.1875	0.5455	
**Liver/ Bile**	0.8125	0.8125	1	1	

**Duct**					
**Lung**	0.2188	0.7955	0.9068	0.9517	
**Lymphoid Leukaemia**					0.4118
**Lymphoma**	0.2727	0.5833	0.7174	0.6087	
**Melanoma**	0	0	0	1	
**Myeloid Neoplasm**					0.2
**Oesophagus**	0.125	0.6471	0.9412	1	
**Other**	0.1818	0.6923	0.6923	0.6923	0
**Ovary**	0.5	0.8	0.871	0.9474	
**Pancreas**	0.6098	0.6098	0.8571	0.9589	
**Plasma Cell Neoplasm**					0.7234
**Prostate**	0.0316	0.0494	0.14	0.8334	
**Sarcoma**	0.4	0.5833	0.5833	0.8571	
**Stomach**	0.1667	0.5	0.8	1	
**Thyroid**	0	0	0	0	
**Urothelial Tract**	0	0	0	1	
**Uterus**	0.1667	0.3	0.7391	1	

Table S6. Dwell Time Estimates.

Dwell times used in this study were from a previous publication by Hubbell and colleagues (2021). We renamed the scenarios so that medium, fast and aggressive-fast dwell times in the previous publication corresponds to slow, medium and fast dwell times in this study.

Cancer Type	Stage	Slow	Medium	Fast

**Anus**	**I**	3	2	1.5
	**II**	1.5	1	0.75
	**III**	0.75	0.5	0.5
	**IV**	0.75	0.5	0.25
**Bladder**	**I**	7	4	2
	**II**	3	2	1
	**III**	1.5	1	0.5
	**IV**	1	1	0.5
**Breast**	**I**	7	4	2
	**II**	3	2	1
	**III**	1.5	1	0.5
	**IV**	1	1	0.5
**Cervix**	**I**	6	4	1.5
	**II**	3	2	0.75
	**III**	1.5	1	0.5
	**IV**	1	1	0.25
**Colon/Rectum**	**I**	3	2	1.5
	**II**	1.5	1	0.75
	**III**	0.75	0.5	0.5

	**IV**	0.75	0.5	0.25
**Gallbladder**	**I**	5	2	1
	**II**	1.5	1	0.5
	**III**	1	1	0.25
	**IV**	0.75	0.5	0.25
**Head and Neck**	**I**	6	4	1.5
	**II**	3	2	0.75
	**III**	1.5	1	0.5
	**IV**	1	1	0.25
**Kidney**	**I**	5	2	1
	**II**	1.5	1	0.5
	**III**	1	1	0.25
	**IV**	0.75	0.5	0.25
**Liver/Bile Duct**	**I**	5	2	1
	**II**	1.5	1	0.5
	**III**	1	1	0.25
	**IV**	0.75	0.5	0.25
**Lung**	**I**	3	2	1.5
	**II**	1.5	1	0.75

	**III**	0.75	0.5	0.5
	**IV**	0.75	0.5	0.25
**Lymphoma**	**I**	6	4	1.5
	**II**	3	2	0.75
	**III**	1.5	1	0.5
	**IV**	1	1	0.25
**Melanoma**	**I**	7	4	2
	**II**	3	2	1
	**III**	1.5	1	0.5
	**IV**	1	1	0.5
**Oesophagus**	**I**	3	2	1.5
	**II**	1.5	1	0.75
	**III**	0.75	0.5	0.5
	**IV**	0.75	0.5	0.25
**Ovary**	**I**	6	4	1.5
	**II**	3	2	0.75
	**III**	1.5	1	0.5
	**IV**	1	1	0.25
**Pancreas**	**I**	5	2	1

	**II**	1.5	1	0.5
	**III**	1	1	0.25
	**IV**	0.75	0.5	0.25
**Prostate**	**I**	5	2	1
	**II**	1.5	1	0.5
	**III**	1	1	0.25
	**IV**	0.75	0.5	0.25
**Sarcoma**	**I**	5	2	1
	**II**	1.5	1	0.5
	**III**	1	1	0.25
	**IV**	0.75	0.5	0.25
**Stomach**	**I**	5	2	1
	**II**	1.5	1	0.5
	**III**	1	1	0.25
	**IV**	0.75	0.5	0.25
**Thyroid**	**I**	5	2	1
	**II**	1.5	1	0.5
	**III**	1	1	0.25
	**IV**	0.75	0.5	0.25

**Urothelial Tract**	**I**	7	4	2
	**II**	3	2	1
	**III**	1.5	1	0.5
	**IV**	1	1	0.5
**Uterus**	**I**	6	4	1.5
	**II**	3	2	0.75
	**III**	1.5	1	0.5
	**IV**	1	1	0.25
**Other**	**I**	7	4	2
	**II**	3	2	1
	**III**	1.5	1	0.5
	**IV**	1	1	0.5

Method S7. Multi-Cancer Early Detection (MCED) Test Sensitivity and Rates of Detection. If the sensitivity of an MCED test for stage I cancer is 20%, then 20% of cancers will be detectable at this stage, and if screening occurs while the cancer dwells in stage I, 20% of cancers will be intercepted before they progress to stage II. No within-stage variation in detectability was modelled; thus, in the model, if a cancer was not detectable at a given stage, it could not be screen detected at that stage, irrespective of the number of screening rounds. Test sensitivity in the model was constrained to increase monotonically with stage. The incremental sensitivity determined the number of additional detectable cancers following stage progression. To continue the example, if test sensitivity at stage II is 50%, an additional 30% of all cancers

will be detectable at this stage (incremental sensitivity). The additional 30% will be intercepted at stage II if screening occurs before the next stage progression.

The proportion of cancers intercepted at each stage are those that are detectable and for which a screening test is performed prior to stage progression. To obtain the proportion of cancers that go through a screening test in the detectable period, evaluation over the dwell time distributions is required (see equations below). In our analysis, we assumed exponential dwell-time distributions. The intercepted fractions were calculated piecewise by stage-at-diagnosis in usual care. Cancers could progress through more than one stage during the annual screening interval; the probability that a cancer developed and proceeded through all stages between screens was dependent on dwell time distribution.

Equations

This information was presented in the supplemental data section of the original interception model (14). To calculate the proportion of incident cancers intercepted, we considered only cancers detected for a positive duration of time (t) prior to clinical diagnosis. The cumulative distribution function for preclinical duration of these cancers is defined as:

$$F(t) = \int_0^t f(x)dx$$

$f(x)$ is the density function of preclinical duration of detectable cancers.

Depending on the screening interval, detectable cancers may present clinically prior to a scheduled screening test. The proportion of interval cancers is given by the expectation:

$$E = \int_0^1 F(t)dt$$

We assumed a screening interval of one year, although the framework is generalisable to other interval lengths (this does not change the limits of integration but the scale of the time axis).

An incident round of screening detects the fraction of cancers that have sufficient preclinical duration and have first become detectable in the interval since the prior screen. The screen-detected fraction is:

$$S_i = \int_0^{\infty} (F(t+1) - F(t))dt$$

Simplifying,

$$= \int_0^{1} \left(1 - F(t)\right)dt$$

For the prevalent round of screening, where the screening interval is effectively indefinitely long, the integral is unrestricted:

$$S_p = \int_0^{\infty} \left(1 - F(t)\right)dt$$

Using the same logic, the probability of a cancer being intercepted at a given stage can be approximated using the cumulative distribution of time in stage instead of the whole detectable time interval.

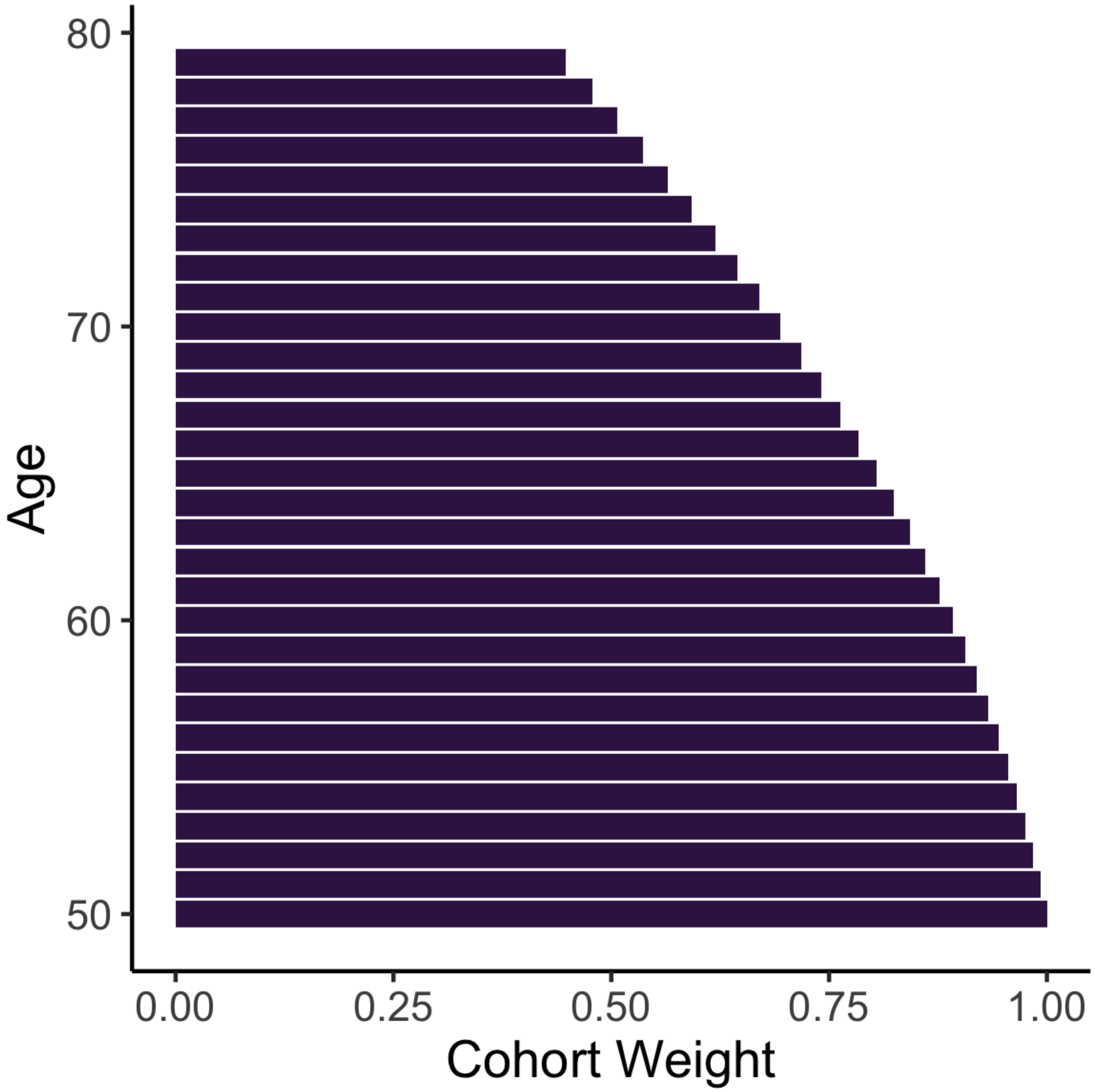

Figure S8. Cohort Weights in the National Screening Programme.

At 50 years of age, 100,000 participants entered the national screening programme and 'aged' through the programme until they were 79 years old. The cohort weights were applied at each age to account for those who have exited the cohort due to death, emigration, or diagnosis with cancer.

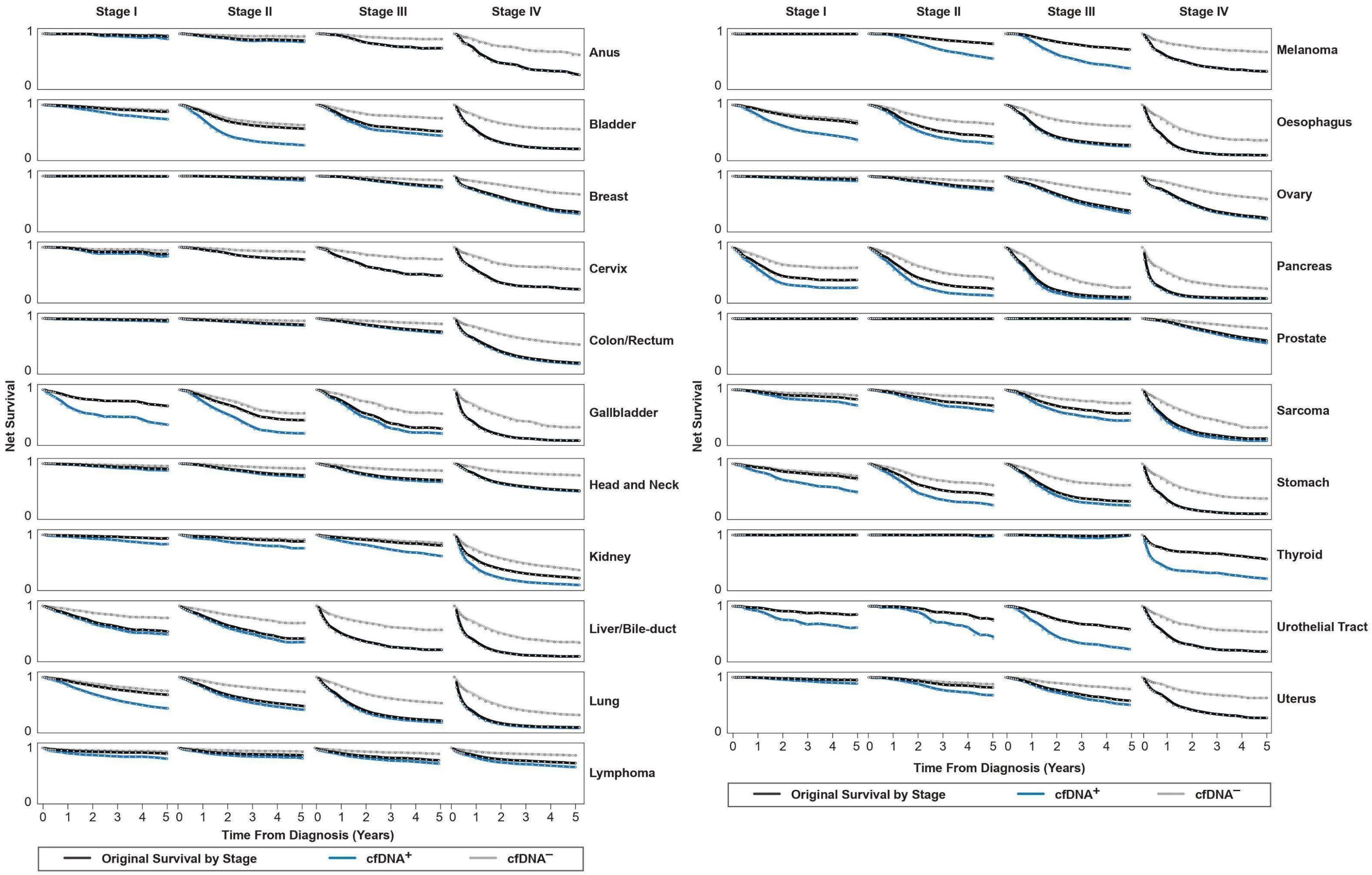

Figure S9. Survival by cfDNA Status and Stage for All Cancer Types Included in This Study.

For each cancer type, the figure shows observed five-year survival data based on cancers diagnosed between 2013 and 2018, as registered by the National Cancer Registration and Analysis Service (NCRAS), and modelled five-year survival data based on cfDNA detectability status (HR = 3) for a person aged 65 years.

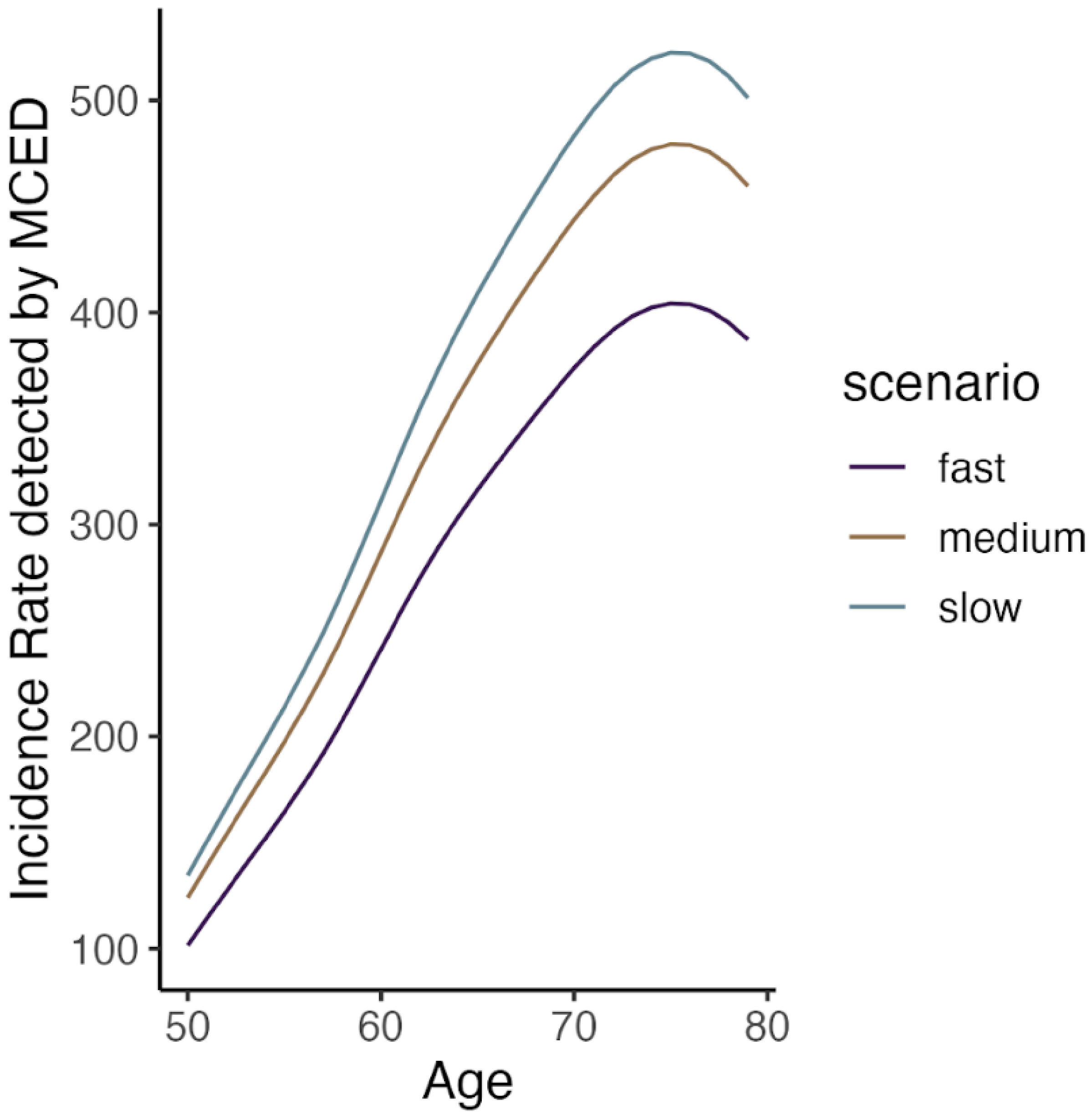

Figure S10. Multi-Cancer Early Detection (MCED) Screen-Detected Cancer Incidence by Age.

The incidence rate for cfDNA detected cancer by age is displayed for each of the dwell time scenarios. Intuitively, the greatest number of cancers are diagnosed in the 'slow' dwell time scenario, because cancers spend more time in each stage, increasing the likelihood they will be screen-detected. The MCED screen-detected incidence rate increases steadily with age, until age 75 years, when it appears to decrease; this reflects the impact of competing causes of mortality.

At older ages, there is greater potential for overdiagnosis, whereby people are at greater risk of dying from another cause before they die from cancer, even if their cancer was clinically significant. Despite this decrease, the incidence rate is still considerable, which suggests that there is the potential for benefit throughout the screening programme.
